# Efficient and stable near-infrared InAs quantum dot light-emitting diodes

**DOI:** 10.1038/s41467-025-57746-1

**Published:** 2025-03-12

**Authors:** Binghan Li, Yu Wang, Jiancheng Zhang, Yaobo Li, Bo Li, Qingli Lin, Ruijia Sun, Fengjia Fan, Zaiping Zeng, Huaibin Shen, Botao Ji

**Affiliations:** 1 Zhejiang Key Laboratory of 3D Micro/Nano Fabrication and Characterization, School of Engineering, Westlake University, Hangzhou, China; 2 Westlake Institute for Optoelectronics, Fuyang, Hangzhou China; 3 Key Laboratory for Special Functional Materials of Ministry of Education, National & Local Joint Engineering Research Center for High-efficiency Display and Lighting Technology, Henan University, Kaifeng, China; 4 Henan International Joint Laboratory of Quantum Dot Materials, School of Materials Science and Engineering, Henan University, Kaifeng, Henan China; 5 CAS Key Laboratory of Microscale Magnetic Resonance and School of Physical Sciences, University of Science and Technology of China, Hefei, China

**Keywords:** Lasers, LEDs and light sources, Quantum dots

## Abstract

Visible quantum dot light-emitting diodes have satisfied commercial display requirements. However, near-infrared counterparts considerably lag behind due to the inferior quality of near-infrared quantum dots and limitations in device architecture suitable for near-infrared electroluminescence. Here, we present an efficient strategy using zinc fluoride to balance ZnSe shell growth across different core quantum dot facets, producing highly regular InAs/InP/ZnSe/ZnS quantum dots with near-unity quantum yield. Moreover, we develop a method of in-situ photo-crosslinking blended hole-transport materials for accurate energy level modulation. The crosslinked hole-transport layers enhance hole transfer to the emitting layer for balanced carrier dynamics in quantum dot light-emitting diodes. The resulting near-infrared quantum dot light-emitting diodes exhibit a peak external quantum efficiency of 20.5%, a maximum radiance of 581.4 W sr^−1^ m^−2^ and an operational half-lifetime of 550 h at 50 W sr^−1^ m^−2^. This study represents a step towards practical application of near-infrared quantum dot light-emitting diodes.

## Abstract (web summary)

Li et al. report ZnF_2_ as the additive to balance shell growth rate of ZnSe in large InAs/InP/ZnSe/ZnS core-multishell quantum dots. An in-situ photo-crosslinking blended hole-transport layer is used to modulate the energy levels for balanced carrier dynamics in NIR LEDs, enabling peak efficiency of 20.5% and operational half-lifetime of 550 h at 50 W sr^−1^ m^−2^.

## Introduction

Solution-processed near-infrared (NIR, 700–1700 nm) light-emitting diodes (LEDs), are cost-efficient to fabricate and compatible with flexible substrates, exhibiting great potential in many applications such as night-vision, telecommunications, and biomedical imaging^1,2^. Among potential emitters, colloidal quantum dots (QDs) stand out for high-performance NIR LEDs due to their wide NIR tunability, bright photoluminescence (PL) and narrow emission linewidths^3,4^. However, NIR QD-LEDs considerably lag behind state-of-the-art visible QD-LEDs, which presents a major obstacle to their widespread application^5^.

To enhance the efficiency of NIR QD-LEDs, different strategies have been developed, including the formation of core/shell QDs and incorporating emitting QDs in wider-gap QD hosts or perovskite^6–9^. So far, NIR QD-LEDs have achieved external quantum efficiencies (EQE) up to 16.98%^10^. However, most of these high-performance NIR QD-LEDs use QDs or matrices containing heavy-metal elements Pb, Cd, and Hg^3,11^, which are severely restricted by the Restriction of Hazardous Substances Directive (RoHS)^12^. While several RoHS-compliant NIR QDs have been explored as emitting materials for QD-LEDs, many of these devices exhibit challenges related to efficiency, radiance, and operational stability (Table 1)^13–16^. Colloidal InAs QDs are particularly promising among RoHS-compliant NIR-emitting QDs, offering tunable excitonic transition from 700 to ~1700 nm through size adjustment^17,18^. Currently, NIR QD-LEDs using InAs-based QDs have reached an EQE of 13.3%^13,19^. However, further improvements are hindered by the lack of advanced synthetic strategy for high-quality InAs-based QDs with finely controlled structures and the limitations on specifically adapted device architecture designs to balance carrier dynamics for efficient NIR electroluminescence (EL).

**Table 1:** Performance summary of high-performance RoHS-compatible NIR LEDs fabricated through a solution process

RoHS-compliant Materials	EQE (%)	Emission peak (nm)	Peak Radiance (W Sr^−1^ m^−2^)	Stability (*T*_50_ (h) @ 50 W Sr^−1^ m^−2^)^a^	Reference
InAs QDs	13.3	1006	2.2	0.2 (*T*_85_)	^13^
Si QDs	8.6	853	~1.63	NA	^14^
AgAuSe QDs	15.8	1046	1.51	NA	^15^
CuInS_2_ QDs	8.1	940	13.3	<0.01	^16^
Sn-Perovskites	3.2	948	226	8.6	^37^
	11.6	898	89	0.68	^38^
Organic radicals	9.6	800	68	NA	^39^
InAs QDs	20.4	905	581.4	550	This work

^a^The half-lifetimes (*T*_50_) at 50 W sr^−1^ m^−2^ are estimated using an empirical equation – ${({R}_{0})}^{{\rm{n}}}$ × ${T}_{50}$ = constant – where ${R}_{0}$ is the initial radiance. An acceleration factor (n) of either 2 or 1.5 is adopted for testing initial radiance larger or smaller than 50 W sr^−1^ m^−2^, respectively, following the principle of overestimating the calculated *T*_50_ at 50 W sr^−1^ m^−2^. Different operational lifetime evaluations (*T*_85_, the time when the radiance drops to 85% of its initial value) are specifically noted. NIR LEDs based on RoHS (Restriction of Hazardous Substances Directive) compliant materials, including tin-based perovskites and organometallic complexes, are also listed in the table.

In this study, we present high-performance NIR QD-LEDs by implementing high-quality heavy-metal-free InAs-based QDs in an electroluminescent device with tailored hole-transport layers (HTLs). Large, regular InAs/InP/ZnSe/ZnS core/multishell QDs with near 100% PL quantum yield (QY) are synthesized using zinc fluoride as a key additive. Employing these QDs as emitters, we find NIR QD-LEDs constructed with benchmark HTLs show only moderate device performance due to unexpected exciton formation in the HTLs, associated with ineffective hole transfer from the HTLs to the narrow-gap QDs. To promote hole transfer, we modulate the energy levels of HTLs by incorporating standard HTL fragments into a photo-crosslinked, rigid matrix of small organic semiconductor molecules with a deep highest occupied molecular orbital (HOMO). This design of QD-LEDs gives a peak EQE of up to 20.5% at 905 nm, a high radiance of 581.4 W sr^−1^ m^−2^ and a long half-lifetime (*T*_50_) of 550 h at 50 W sr^−1^ m^−2^.

## Results

### Synthesis and properties of InAs-based quantum dots

**Fig. 1:**
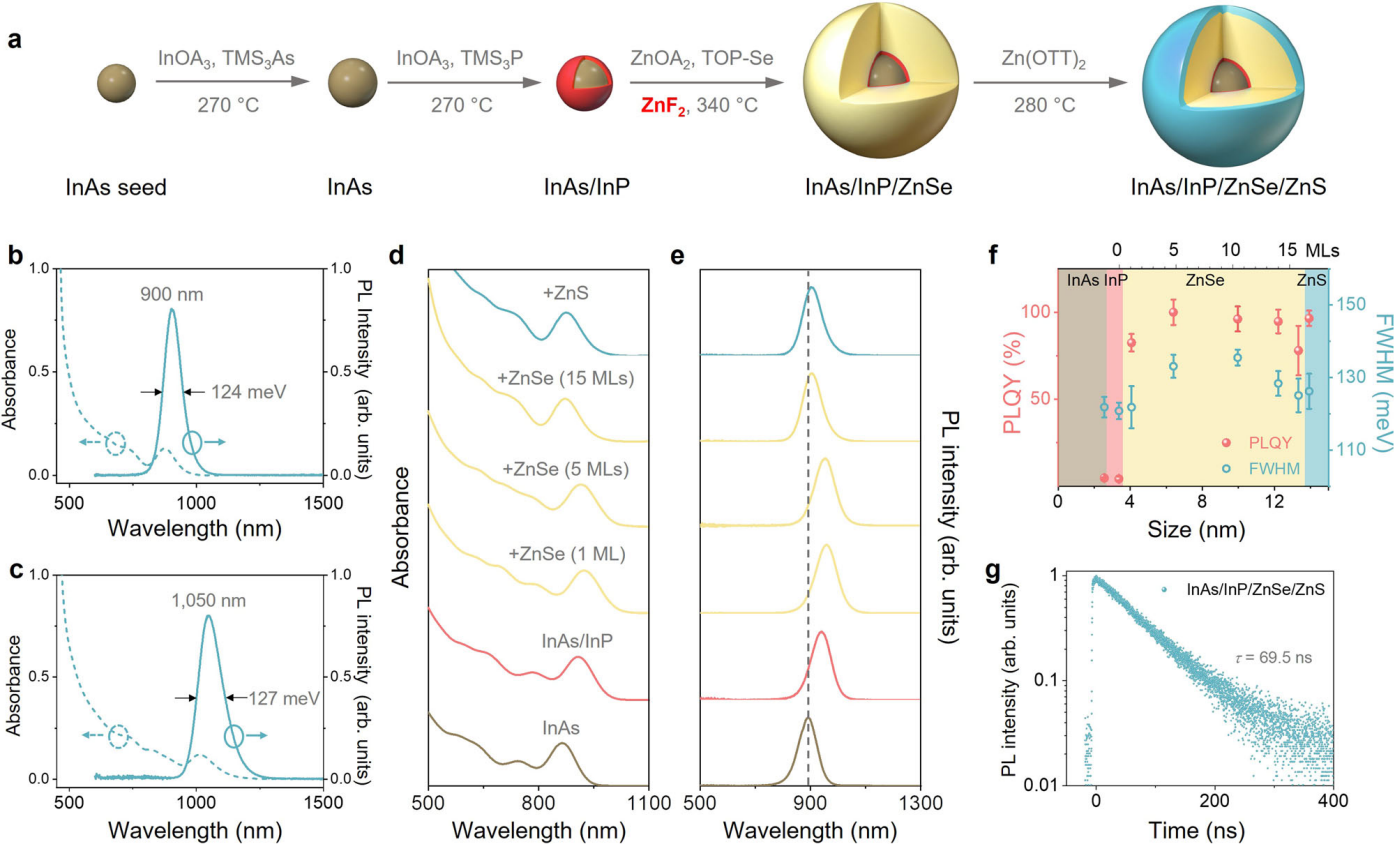
Synthesis and optical characterizations of large InAs/InP/ZnSe/ZnS core/multishell QDs. **a** Scheme of the protocol for synthesizing InAs/InP/ZnSe/ZnS quantum dots (QDs). The InAs core and InP shell are synthesized at 270 °C by reacting indium oleate (InOA_3_) with tris(trimethylsilyl)arsine ((TMS)_3_As) and tris(trimethylsilyl) phosphine ((TMS)_3_P), respectively. The ZnSe shell grows with zinc oleate (ZnOA_2_) and trioctylphosphine selenide (TOPSe) at 340 °C, while the ZnS shell is formed with zinc octanethiolate (Zn(OTT)_2_) at 270 °C. ZnF_2_ is added during ZnSe shell growth to promote isotropic morphology. Absorption (dash line) and photoluminescence (PL) spectra (solid line) of InAs/InP/ZnSe/ZnS QDs with PL peaks at 900 nm (**b**) and 1050 nm (**c**). Evolution of absorption spectra (**d**), PL spectra (**e**), PL quantum yield (PLQY, light coral) and full width at half maximum (FWHM, sky blue) (**f**) of the QDs shown in (**b**) during the overcoating reaction. The dashed line in (**e**) is inserted as a guide to highlight the shifts in the PL peaks. The error bars of PLQYs and FWHMs in (**f**) represent the standard deviation of multiple syntheses results with respect to the mean values. **g** Ensemble PL decays of the final QDs sample.

From a QD engineering point of view, large QDs with elaborate passivation shells are ideal emitters because this architecture is beneficial to mitigate non-radiative decay caused by interparticle energy transfer in QDs films^20^, to facilitate the formation of electrogenerated excitons in the QDs^21^, and to reduce non-radiative Auger recombination for the suppression of EQE roll-off^22^. However, previous studies on InAs-based QDs have typically reported small sizes and PLQYs below 80%^23–26^. The synthesis of large InAs-based QDs (>10 nm) with higher PLQY remains a critical challenge. To address this, we develop an effective strategy to produce large, high-quality InAs-based QDs with InAs cores overcoated with an InP buffer layer, a ZnSe passivation layer, and a ZnS terminating layer (Fig. 1a). This graded shell construction approach enables a progressive confinement of exciton wavefunctions in the InAs core with a decreased sequential lattice mismatch, which suppresses the formation of compressive strain-induced interfacial defects (see Methods and Supplementary Figs. 1, 2).

Uniform InAs cores were produced by adding amorphous InAs clusters on pre-formed InAs seeds until the target size was reached^27^, at which point InP was deposited, producing InAs/InP core/shell QDs. The purified InAs/InP QDs were subsequently reacted with Zn and Se precursors at 340 °C to deposit ZnSe shells, during which a key inorganic additive – ZnF_2_ – was introduced to control shell growth. Finally, a thin ZnS layer was grown by decomposing Zn-thiolate at 280 °C, followed by a surface treatment to exchange thiolate with carboxylate ligands (Supplementary Fig. 3)^28^. The overall QDs size was precisely controlled by regulating the ZnSe thickness.

Figure 1b shows the absorption and PL spectra of a large InAs-based QD sample (average size, ~13.5 nm, termed QDs-900), showing a PL peak at ~900 nm, with a PLQY approaching 100% and a full-width at half-maximum (FWHM) of 124 meV. To tune the emission wavelength, larger InAs cores were synthesized, on which multiple layers were then deposited through the developed procedure (Supplementary Fig. 4, 5). This produced QDs (~13 nm) emitting at 1050 nm in the short-wavelength infrared range with near-unity PLQY (Fig. 1c and Supplementary Fig. 6), validating the practical applicability of our approach for synthesizing tunable NIR InAs-based QDs of exceptional quality.

To track the shell growth reaction, we measured the evolving absorption and PL spectra during the synthesis of QDs-900 (Fig. 1d, e and Supplementary Figs. 7, 8). Both the absorption features and the PL peaks of the InAs cores continuously shift towards the red during the growth of InP (~1 monolayer (ML)) and ZnSe (3 MLs) due to exciton delocalization. Intriguingly, further ZnSe shell growth induce a gradual blueshift of excitonic absorption and PL peaks. The InAs cores were compressively deformed as they adapted to the thick, epitaxial growth of ZnSe, resulting in enlarged bandgaps and blue-shifting of spectra^29^. The absorption features remained well-resolved and PL spectra remained narrow throughout the overcoating process, indicating homogeneous shell growth. Although InP deposition did not have a significant influence on the PLQY (<10% for InAs and InAs/InP QDs), a drastic increase in the PLQY to near 100% was observed when a thin ZnSe layer (~3 MLs) was overcoated. Near-unity PLQY was preserved as the ZnSe layer was increased to 14 MLs but decreased to ~78% with the coating of an additional 2 MLs of ZnSe, which was, recovered after deposition of the protective ZnS layer (Fig. 1f). The QDs-900 exhibited monoexponential PL decay dynamics, consistent with their near-unity PLQY (Fig. 1g).

### Effects of ZnF_2_ on structural properties of quantum dots

**Fig. 2:**
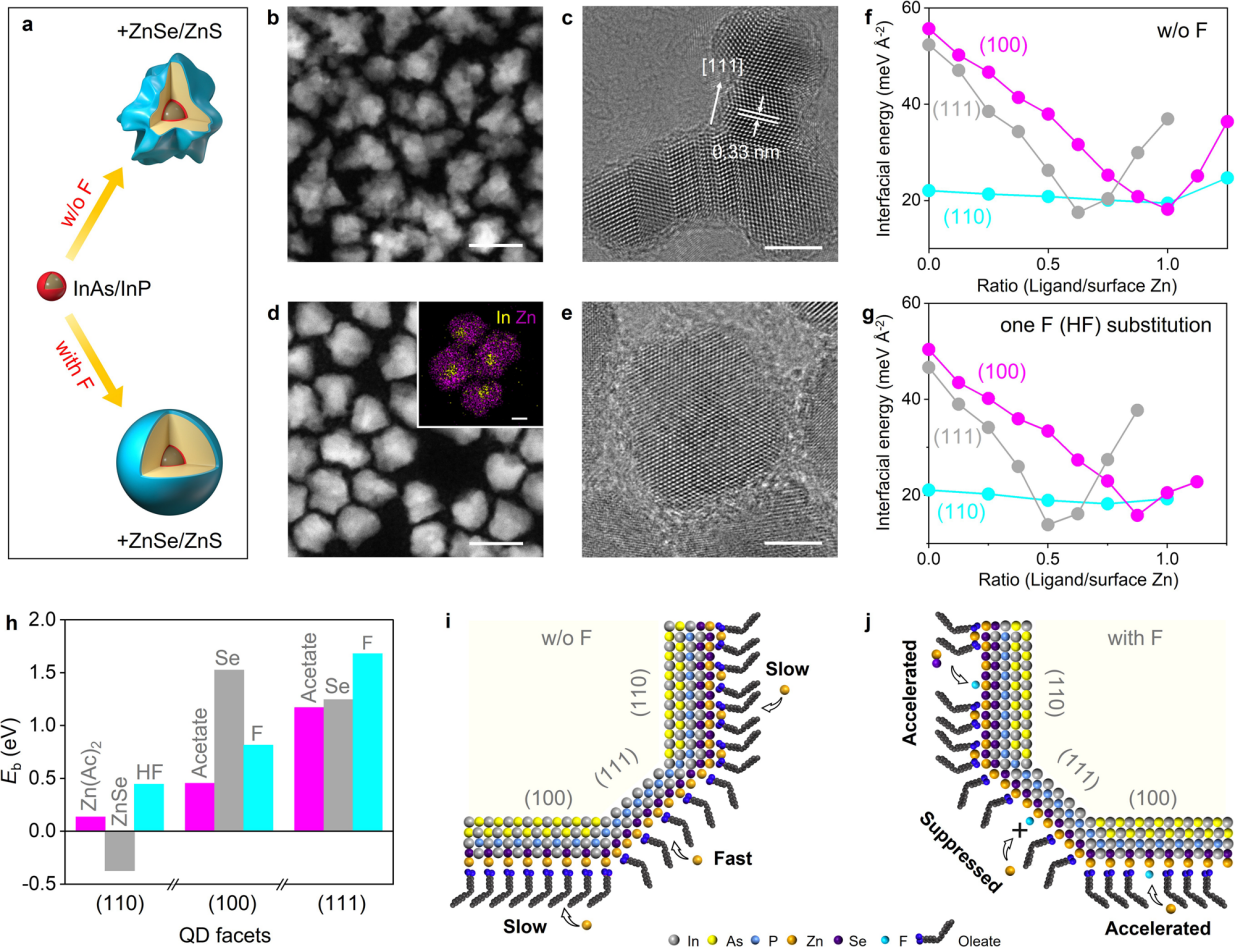
Impact of inorganic ZnF_2_ on the shell morphology of large InAs/InP/ZnSe/ZnS core/multishell QDs. **a** Schematic illustrating ZnSe shell growth during synthesis without ZnF_2_ (top) and with ZnF_2_ (bottom). **b**, **c** STEM and HRTEM images of QDs synthesized without ZnF_2_. **d**, **e** STEM and HRTEM images of QDs synthesized with ZnF_2_. The inset in (**d**) shows the corresponding energy dispersive spectroscopy (EDS) elemental mapping of In (yellow) and Zn (purple). The scale bars in the STEM and HRTEM images and the EDS mapping inset are 20 nm, 5 nm, and 5 nm, respectively. **f**, **g** Interfacial energies of the {100}, {111}, and {110} facets using a slab model consisting of an InAs substrate with a single epitaxial ZnSe monolayer as a function of ligand coverage: **f** without fluoride species and **g** with one original ligand replaced by F or HF. **h** Binding energies (*E*_b_) of various species on the {100}, {111} and {110} facets at optimal ligand coverage. **i** Schematic diagram depicting preferential ZnSe growth on the {111} facet without ZnF_2_, leading to anisotropic morphology. **j** Schematic diagram of QD growth with ZnF_2_ illustrating the critical role of fluoride species in reducing growth rate differences across the three facets and thus promoting isotropic ZnSe shell growth. The gray, yellow, light blue, orange, dark purple, cyan, navy blue, and black spheres represent the elements In, As, P, Zn, Se, F, O, and C, respectively.

We find ZnF_2_ played a crucial role in regulating ZnSe shell growth (Fig. 2a). InAs/InP/ZnSe/ZnS QDs synthesized without ZnF_2_ (referred to as untreated QDs) exhibited highly irregular shapes (Fig. 2b, Supplementary Fig. 9). High-resolution transmission electron microscopy (HRTEM) image reveals preferential shell growth on the {111} planes of the QDs, leading to a polypod-like morphology (Fig. 2c). These untreated QDs display a lower PLQY of ~82%, possibly due to trap states caused by their irregular morphology. By contrast, the QDs-900 produced with ZnF_2_ exhibit regular morphology with tight size distributions at all stages of shell growth (Fig. 2d and Supplementary Figs. 10, 11). Element mapping clearly identify a core/shell architecture with In and Zn atoms distributed separately in the inner and outer regions. High crystallinity and well-defined lattice fringes are verified by X-ray diffraction (XRD) and HRTEM, respectively (Fig. 2e and Supplementary Fig. 12).

We attribute the uniform morphology of ZnF_2_-treated QDs to in situ generated HF. ZnF_2_ is an easily handled inorganic salt, insoluble in the reaction solvent. At high temperatures, ZnF_2_ gradually reacts with oleic acid, producing HF slowly and continuously^30^. This steady production of HF maintains a consistent concentration of fluoride species throughout the synthesis. Syntheses using hydrofluoric acid instead of ZnF₂ produced irregular QDs, ruling out surface oxides as the cause of irregular morphology (Supplementary Fig. 13). This also highlights the necessity of continuous fluoride presence, considering hydrofluoric acid tends to deplete quickly early in the shell growth process.

To understand how fluoride species promote isotropic ZnSe shell morphology, we studied the surface structure and energetics of InAs-based core/shell QDs coordinated with carboxylate ligands using first-principles DFT simulations. A slab model of an InAs substrate, covered by a single epitaxial monolayer of ZnSe (Supplementary Fig. 14), was employed to calculate the interfacial energies of three low-index facets: the polar {100} and {111} facets, and the nonpolar {110} facet, as identified by XRD patterns of InAs QDs. We have confirmed that neglecting the thin InP layer does not qualitatively affect the results while maintaining computational efficiency (Supplementary Fig. 15). Two types of ligands, acetate and zinc acetate (ZnAc_2_), were employed as representative models of long-chain ligands to coordinate with the polar zinc-rich {100} and {111} facets, as well as the nonpolar {110} facet. Figure 2f, g illustrate how the interfacial energies of these facets vary with ligand coverage in the absence or presence of fluoride species.

As shown in Fig. 2f, the {100} and {110} facets display the lowest interfacial energies at full ligand coverage (one acetate or 0.5 ZnAc_2_ per Zn atom) without fluoride. For the {111} facet, interfacial energy decrease to a minimum at around 0.625 acetate per Zn atom, then rises as coverage increases, suggesting the {111} facet maintains lower ligand density compared to the {100} and {110} facets when achieving minimal interfacial energy. The ligand binding configuration under the optimal ligand coverage for these three facets is shown in Supplementary Fig. 16. Binding energy calculations reveal acetate forms stronger coordination bonds on the {111} facet than on the {100}, while Zn(Ac)₂ bonds are weakest on the {110} (Fig. 2h). Additionally, We analyze the binding energies of Se on the {100} and {111} facets, and ZnSe on the {110} facet to assess surface reactivity and growth rates. We find that Se binds as strongly as acetate on the {111} facet but much more strongly on the {100} facet, suggesting Se can more effectively displace ligands on the {100} facet. As the HRTEM analysis reveals the {111} facet exhibits the fastest growth, this indicates ligand density plays a predominant role in determining growth rates. Conversely, the weaker binding of ZnSe on the {110} facet implies a slower growth rate along the [110] direction.

Next, we examine how in-situ generated fluoride species affect interfacial energies by analyzing ligand coverage changes when a single F or HF molecule was coordinated, considering the low concentration of fluoride species in the synthesis (Fig. 2g). Stronger binding energies of F or HF on all facets support this ligand exchange (Fig. 2h), which is also experimentally confirmed by precipitation when excess hydrofluoric acid reacted with the InAs-based QDs. The binding energy studies indicate that fluoride species replace one original ligand on each facet, thus reducing overall ligand density. On the {100} facet, Se retains a stronger affinity than F, and the reduced carboxylate coverage due to smaller F accelerates growth. Conversely, on the {111} facet, F binds with an energy of ~1.6 eV, significantly stronger than both acetate and Se, making this facet less accessible for shell growth. The exclusion of F⁻ incorporation into the QD lattice is confirmed by the low molar ratio of F⁻ ions to QDs (Supplementary Fig. 17). On the nonpolar {110} facet, HF binds with moderate strength, which reduces ligand density without significantly impeding growth. Overall, fluoride species help balance growth rates across the three facets, resulting in a more uniform QD morphology. The schematics of ZnSe shell growth without and with ZnF_2_ are displayed in Fig. 2i, j, respectively. To confirm this effect, we conducted control experiments by introducing ZnF₂ after the ZnSe shell reached a thickness of 5 MLs. The resulting QDs display regular morphology with near-unity PLQY (Supplementary Fig. 18). All these results unambiguously indicate the utilization of inorganic ZnF_2_ is essential for synthesizing regular-shaped, large InAs-based NIR QDs with superior PL properties.

### Near-infrared quantum dot light-emitting diodes

**Fig. 3:**
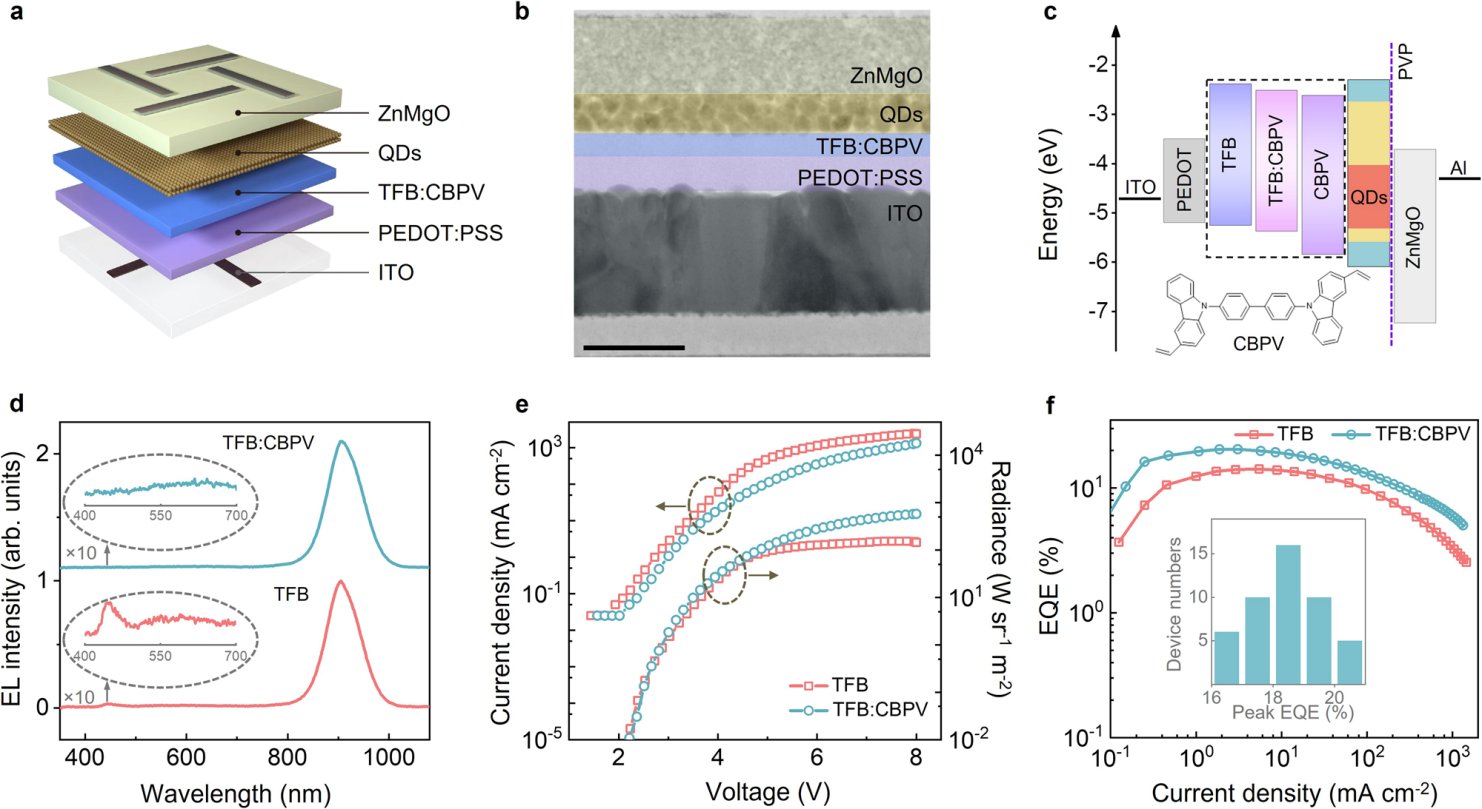
Comparative performance of NIR QD-LEDs based on TFB and crosslinked TFB:CBPV hole transport layers (HTLs). The scheme (**a**) and cross-sectional TEM image (**b**) of the QD-LEDs with direct structure. The scale bar in (**b**) is equal to 50 nm. The thicknesses of the ZnMgO layer (electron transport layer), QD layer, crosslinked poly(9,9-dioctylfluorene-co-N-(4-(3-methylpropyl))-diphenylamine:4,4′-bis(3-vinyl-9H-carbazol-9-yl)−1,1′-biphenyl (TFB:CBPV, HTL) and poly(ethylenedioxythiophene):polystyrene sulfonate (PEDOT:PSS, hole injection layer) were 38, 20, 12 and 17 nm, respectively. **c** Flat energy level diagram of QD-LEDs. The highest occupied molecular orbital (HOMO) and lowest-unoccupied molecular orbital (LUMO) energy levels of various functional layers were obtained using UPS and optical measurements. The conduction band (CB) and valence band (VB) energy levels of InAs/InP core QDs, bulk ZnSe and ZnS are shown in red, yellow and sky blue, respectively. Inset: the molecular structure of CBPV. **d** EL spectra of NIR QD-LEDs using TFB (light coral) and crosslinked TFB:CBPV (sky blue) as HTLs. The applied voltage was 4.0 V. Evident parasitic emission from the HTLs can be observed in the TFB-based devices (light coral, inset), which was effectively suppressed in TFB:CBPV-based devices (sky blue, inset). **e** Current density and radiance as a function of voltage for NIR QD-LEDs based on TFB HTLs (light coral) and TFB:CBPV HTLs (sky blue). **f** External quantum efficiency (EQE) as a function of voltage for NIR QD-LEDs based on TFB HTL (light coral) and TFB:CBPV HTL (sky blue). The inset shows corresponding peak EQE histograms of more than 45 QD-LEDs based on TFB:CBPV HTLs.

Next, we fabricated NIR QD-LEDs with a direct structure composed of as-synthesized QDs sandwiched between an electron transport layer (ETL) of ZnMgO nanocrystals and an HTL of organic semiconductor polymer (Fig. 3a–c). QDs-900 were selected as the emitting layer, given that the non-radiative Förster resonance energy transfer (FRET) in a closely packed QD film can be alleviated by increasing interparticle distances^31^. Indeed, the QDs-900 film exhibits considerably lower FRET efficiency (33.1%) than small QD (~6.8 nm) films (64.1%) (Supplementary Figs. 19, 20). An ultrathin insulating layer of polyvinylpyrrolidone (PVP) was inserted between the QDs and the ZnMgO layers, avoiding PL quenching by preventing interlayer charge transfer (Supplementary Fig. 21)^32^.

A standard HTL material – poly(9,9-dioctylfluorene-alt-N-(4-s-butylphenyl)-diphenylamine) (TFB) – was used in construction of the QD-LEDs. These TFB-based QD-LEDs display NIR EL with a moderate peak EQE of 14.2% and a maximum radiance of 154 W sr^−1^ m^−2^ (Fig. 3d–f). However, closer observations of the EL spectrum reveal discernible parasitic emission from the TFB, indicating a considerable number of excitons had formed in TFB^33^. This is unexpected, as almost no exciton transfer is anticipated from the QDs to the TFB given that the bandgap energy of the QDs is much smaller than that of the TFB. This is verified by the fact that transient PL lifetimes are almost identical irrespective of whether the QD films were deposited on glass or on TFB (Supplementary Fig. 22). We thus ascribe the formation of excitons in TFB to electron transfer from the QD layer to the TFB. This current leakage is surprising, considering the seemingly large energy offset between the conduction band of the core QDs and the lowest-unoccupied molecular orbital (LUMO) of the TFB (Fig. 3c and Supplementary Fig. 23). Our attempt to employ a TFB-derived hole-transport polymer with both a shallower LUMO and HOMO to block the electron leakage result in QD-LEDs with more severe parasitic emission from the HTLs (Supplementary Fig. 24). We therefore conclude that the low efficiency of hole transfer from TFB to the QDs, probably due to the deep valence band of NIR QDs, causes hole accumulation in TFB, lowering the barrier for electron leakage at the QD/HTL interface.

To improve hole transfer efficiency in the device, we tailored our strategy of in-situ photo-crosslinking blended hole transport materials without photo-initiator to precisely modulate the energy levels of HTLs. Specifically, a conjugated olefin-based, small-molecule hole transport material with a deeper HOMO energy level than TFB – 4,4′-bis(3-vinyl-9H-carbazol-9-yl)-1,1′-biphenyl (CBPV, the molecular structure is shown in Fig. 3c) – was blended with TFB to form a uniform HTL film. After annealing and irradiation with mild ultraviolet (UV) light, the crosslinking reaction of styrene moieties in the CBPV was stimulated to further enhance the TFB/CBPV interaction. The formation of a crosslinked CBPV matrix was verified by the enhanced solvent resistance (Supplementary Fig. 25). While the effective optical bandgaps of TFB and the crosslinked TFB:CBPV HTLs remained almost equivalent, we managed to progressively deepen both the HOMO and LUMO onset energies of the crosslinked HTL by increasing the weight ratio of CBPV/TFB (Supplementary Fig. 23). The HOMO and LUMO onset energies of the crosslinked HTLs are estimated to be ~0.12 eV deeper than those of TFB when 20 wt% CBPV was employed (Fig. 3c). Considering that the QDs have much larger dimensions than either the conjugated TFB fragments or the CBPV molecules, every QD is expected to directly interact with both ingredients in the crosslinked HTL, further facilitating hole transfer from the HTL to negatively charged QDs and promoting radiative recombination between electrically injected holes and electrons in the emitting QD layer^21,33^.

Employing the photo-crosslinked HTL with 20 wt% CBPV, the QD-LEDs exhibit pure QD-associated NIR EL without any detectable parasitic emissions, indicating substantially reduced exciton formation in the HTL (Fig. 3d and Supplementary Fig. 26). To verify whether the suppression of current leakage was a result of improved hole transport at the HTL/QDs interface, we measured the voltage-dependent capacitance (CV) characteristics of the QD-LEDs using TFB or TFB:CBPV with different weight ratios as HTLs (Supplementary Fig. 27). Compared with TFB-based QD-LEDs, increasing the CBPV/TFB ratio in HTLs result in a gradual decrease in capacitance peak value at lower bias, indicating more effective hole transfer and reduced hole accumulation in the HTLs^34^. However, further increasing the CBPV weight ratio to 50% leads to the reappearance of parasitic emissions from the HTL, attributed to inefficient hole transport due to the lower hole mobility of CBPV (~10^−4 ^cm^2 ^V^−1^ s^−1^ compared to 10^−3 ^cm^2 ^V^−1^ s^−1^ for TFB) (Supplementary Fig. 26). To validate this, we fabricated QD-LEDs using poly(9-vinylcarbazole) (PVK) as the HTL, which has a deep HOMO level but significantly lower hole mobility than TFB. These QD-LEDs also exhibit non-negligible parasitic emissions from PVK and a capacitance peak at high bias in CV curve (Supplementary Fig. 28)^35^. These results indicate an appropriate TFB/CBPV ratio is critical to promote hole transfer at the HTL/QDs interface while maintaining high hole mobility for efficient hole transport within the HTL under low bias. Thereafter, 20 wt% of CBPV was selected for the photo-crosslinked HTL.

As shown in Fig. 3e, compared to TFB-based QD-LEDs, TFB:CBPV-based QD-LEDs have lower current densities at the same voltage due to the eliminated electron leakage and decreased hole mobility of the photo-crosslinked HTL^22,33,36^, but they display similar radiance at low voltage and a much higher radiance at high voltage. Remarkably, these TFB:CBPV-based QD-LEDs exhibit a peak EQE of 20.5% at a driving voltage of 3.3 V (current density, 3.1 mA cm^−2^; radiance, 5.47 W sr^−1^ m^−2^) and a maximum radiance of 581.4 W sr^−1^ m^−2^ (Fig. 3e, f). These values, to our best knowledge, exceed the performance of current state-of-the-art RoHS-compatible NIR QD-LEDs (Supplementary Fig. 29 and Table 1)^37–39^. A high EQE of >10% is maintained across a wide range of current densities (0.15–244 mA cm^−2^), corresponding to a radiance range of 0.13–216 W sr^−1^ m^−2^. The excellent reproducibility of high-performance QD-LEDs based on TFB:CBPV HTLs is demonstrated by the EQE histograms for >45 devices, showing an average EQE of ~18.5% with low deviation (inset in Fig. 3f). We sent our devices to National Institute of Metrology of China for certification. These devices exhibited a certified peak EQE of 19.0%, closely equivalent to the average peak EQE obtained in our laboratory (Supplementary Fig. 30). Note that employing the photo-crosslinked HTL also enhances the performance of QD-LEDs using untreated QDs, increasing the peak EQE from 8.5% to 9.8% (Supplementary Fig. 31). Compared to the QDs-900 film, the untreated QDs film display much lower PL intensity due to the presence of more defective QDs, which act as non-radiative recombination centers in dense QDs film (Supplementary Fig. 32). This causes the inferior performance of QD-LEDs using untreated QDs, highlighting the critical role of near-unity-emitting QDs achieved with the key inorganic additive ZnF_2_.

### Device stability measurements

**Fig. 4:**
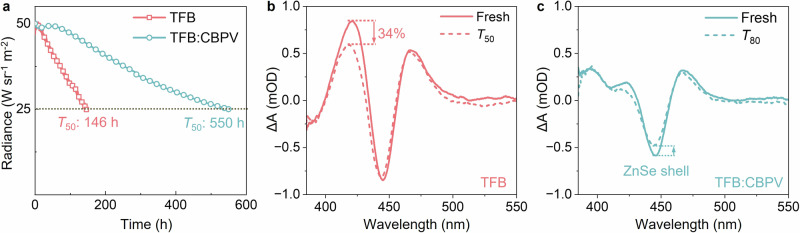
Operational stability tests of QD-LEDs using TFB and TFB:CBPV as HTLs. **a** Operational half-lifetime (*T*_50_) measurements of QD-LEDs based on TFB (light coral) and crosslinked TFB:CBP (sky blue) HTLs. The measurements were performed at constant driving current densities, corresponding to an initial radiance of 50 W sr^−1^ m^−2^. Electrical pump transient absorption (ETA) spectra of QD-LEDs using TFB (**b**) and photo-crosslinked TFB:CBPV (**c**) as HTLs, respectively, before (solid line) and after degradation (dash line). The degradation of QD-LEDs was accelerated under a constant driving voltage of 5 V. During these degradation assessments, TFB-based QD-LED exhibited a ~50% decrease in the radiance (R_0_ = 100 W sr^−1^ m^−2^), while TFB:CBPV-based QD-LED exhibited a ~20% decrease (R_0_ = 125 W sr^−1^ m^−2^). ETA characterization conditions: voltage, −5 V; frequency, 1 kHz; electric pulse width, 10 µs; white light probing frequency, 2 kHz.

To evaluate the general stability of the QD-LEDs, we measured the operational lifetimes of both devices encapsulated in UV-curable resin. As shown in Fig. 4a, the TFB-based QD-LEDs show a *T*_50_ half-lifetime (the time when the radiance drops to 50% of its initial value) of ~146 h at an initial radiance of 50 W sr^−1^ m^−2^. In contrast, the TFB:CBPV-based QD-LEDs exhibit *T*_95_ and *T*_50_ lifetimes of ~94 h and ~550 h at an initial radiance of 50 W sr^−1^ m^−2^, respectively. The unprecedented operational lifetimes of our devices place them among high-performance solution-processed RoHS-compatible NIR-LEDs (Table 1 and Supplementary Table 1). To elucidate the origin of the enhanced stability of QD-LEDs with the crosslinked HTL, we applied electrical pump transient absorption spectroscopy (ETA) to measure the change of Stark signals induced by the electric field in both types of QD-LEDs before and after degradation^40^. As shown in Fig. 4b, TFB-based QD-LED exhibits a decrease of ~34% in the TFB Stark signal at ~420 nm, while the Stark signal of the ZnSe shell of QDs at 445 nm displays minimal change, indicating the degradation of TFB. We believe that hole accumulation in TFB due to ineffective hole transfer across the QDs/TFB junction causes permanent oxidation of the TFB, resulting in device degradation^41^. By contrast, TFB:CBPV-based QD-LED shows no substantial change in the Stark signal of HTL at ~393 nm, an indication of undegraded HTL (Fig. 4c). However, a ~21% decrease in the ZnSe shell Stark signal suggest the device aging mainly originated from the QDs degradation. Therefore, we conclude that the enhancement in device stability is attributed to the reduced hole accumulation in HTL and enhanced stability of the photo-crosslinked HTL.

In summary, we successfully construct high-performance NIR QD-LEDs by combining advanced synthesis with rational device design. An efficient strategy is developed to synthesize high-quality NIR-emitting InAs/InP/ZnSe/ZnS QDs with symmetrical morphology. The key to success is the persistent in situ generation of HF throughout the synthesis by introducing ZnF_2_, kinetically homogenizing shell growth. We develop a tailored HTL – embedding TFB into a photo-crosslinked matrix of CBPV molecules, which significantly improved the hole transfer efficiency. This results in high-performance NIR QD-LEDs with a high EQE of 20.5%, high radiance, and long stability, highlighting the considerable commercial potential of these solution-processed devices. Our synthetic strategy will provide an ideal model for further in-depth fundamental studies of NIR QDs. We envision photo-crosslinking of HTLs is highly compatible with flexible substrates or mass production of patterned QD-LEDs through photo-lithography techniques.

## Methods

### Chemicals

Indium acetate (In(Ac)_3_, 99.99%), zinc acetate (Zn(Ac)_2_, 99.99%), selenium (99.99%), palmitic acid (PA, 99%), di-n-octylamine (DOA, 97%), squalane (96%) and polyvinylpyrrolidone (PVP, average M_w_, ~29,000) were purchased from Sigma-Aldrich. Zinc fluoride (ZnF_2_, 99%), 1-octanethiol (OTT, 98.5%) and 1-octadecene (ODE, 90%) were purchased from J&K Scientific. Oleylamine (OAm, 80–90%) and n-octane (99%) were purchased from Acros Organics. Tri-n-octylphosphine (TOP, 97%) was purchased from Strem Chemicals. Oleic acid (OA, 99%) was purchased from Psaitong. Poly(9,9-dioctylfluorene-co-N-(4-(3-methylpropyl))-diphenylamine) (TFB) was purchased from American Dye Source. Poly(ethylenedioxythiophene):polystyrene sulfonate (PEDOT:PSS) was purchased from Heraeus Deutschland. 4,4′-bis(3-vinyl-9H-carbazol-9-yl)-1,1′-biphenyl (CBPV) was purchased from Suzhou Oupuke Display Technology. Toluene, ethanol, hexane and acetone were extra-dry grade. Tris(trimethylsilyl)phosphine ((TMS)_3_P) and Tris(trimethylsilyl)arsine ((TMS)_3_As) were synthesized by reacting trimethylsilyl chloride with Na/K phosphide and Na/K arsenide, respectively^42^. The solvents of TOA, ODE and squalene were degassed at 120 °C for 1 h and stored in an N_2_-filled glovebox.

### Preparation of precursors

InAs cluster precursor: a mixture of 876 mg of In(Ac)_3_, 2.54 g of OA, 258 mg of InF_3_ and 15 mL of squalane was degassed at 120 °C in a three-neck flask under vacuum. 1.5 mmol of (TMS)_3_As was mixed with 3.0 mL of squalane and 4.5 mmol of DOA and annealed at 50 °C for 1 h in an N_2_-filled glovebox. These two solutions were cooled to room temperature and mixed to prepare the InAs cluster precursor. InP cluster precursor: a 0.2 M In(PA)_3_ solution was obtained by mixing 2.63 g of In(Ac)_3_, 6.92 g of PA, 10.0 g of TOP and 27 mL of ODE in a three-neck flask, followed by degassing at 120 °C under vacuum for 1 h. The InP cluster precursor was prepared by mixing 3.1 mL of In(PA)_3_ solution, 3.1 mL of ODE and 90 μL of (TMS)_3_P in a N_2_-filled glovebox at room temperature. Zn(OA)_2_ precursor: 11.0 g of Zn(Ac)_2_, 38 mL of OA and 112 mL of TOA were loaded in a three-neck flask. The solution was then degassed at 120 °C under vacuum for 1 h. TOP-Se precursor: a 0.4 M TOP-Se solution was prepared by dissolving 2.37 g of selenium powders in 15 mL of TOP and 60 mL of TOA in an N_2_-filled glovebox. ZnF_2_ precursor: 100 mg of ZnF_2_ were added in 2.0 mL of TOA to form a suspending solution.

### Synthesis of InAs/InP core/shell quantum dots

In a typical synthesis of InAs seeds, 292 mg of In(Ac)_3_, 0.847 g of OA, 86 mg of InF_3_ and 5.0 mL of ODE were mixed in a three-neck flask and degassed at 120 °C under vacuum for 1 h. Meanwhile, an arsenic precursor mixture of 0.5 mmol (TMS)_3_As, 1.0 mL of ODE and 1.5 mmol of DOA was stirred at 50 °C for 1 h in an N_2_-filled glovebox. The flask was heated to 300 °C under N_2_ flow, followed by the quick injection of the arsenic precursor under vigorous stirring. The temperature was maintained at 300 °C for 30 min and then cooled to 270 °C. The InAs cluster precursor was then added dropwise through a syringe pump at a rate of 4.2 mL h^−1^ until the exciton absorption peak of InAs QDs reached 865 nm. Subsequently, the InP cluster precursor was injected into the flask at a rate of 3.5 mL h^−1^. When the first absorption peak was located at ~900 nm, the crude solution was cooled down to room temperature and transferred to the glovebox. The InAs/InP QDs were purified by ethanol and toluene for multiple times and redispersed in toluene with an optical density of 0.7 at 500 nm. To produce QDs that emit at longer wavelength, more InAs cluster precursor (~18 mL) together with 1.9 mL of OA and 170 mg of InF_3_, were added to shift the first absorption peak of InAs QDs to ~1080 nm. In the following, InP cluster precursor was injected at a rate of 1.8 mL h^−1^ until the first absorption peak reached 1160 nm. The purified InAs/InP QDs were dissolved in toluene with an optical density of 1.4 at 500 nm. Note that during the synthesis of InAs and InAs/InP QDs, the addition of InF_3_ had minimal impact on the morphology and optical properties of the final InAs/InP/ZnSe/ZnS QDs.

### Synthesis of InAs/InP/ZnSe quantum dots

A mixture of 6.0 mL of TOA, 0.12 g of Zn(Str)_2_, and 20 mg of ZnF_2_ was degassed in a three-neck flask at 120 °C under vacuum for 1 h. Under N_2_ flow, 1.0 mL of InAs/InP QDs solution was added and then the flask was heated to 340 °C in 15 min. The ZnSe shell growth was started by adding 1.6 mL of Zn(OA)_2_ (0.4 M) and 1.5 mL of TOP-Se (0.4 M) precursors dropwise in sequence and maintained for 30 min. The thickness of ZnSe can be easily adjusted by the amount of ZnSe precursors. 0.2 mL of ZnF_2_ precursor was introduced in the flask every two additions of ZnSe precursors. A typical synthesis of large InAs/InP/ZnSe QDs with an emission peak at ~900 nm requires six repetitions of ZnSe precursors injection.

### Synthesis of InAs/InP/ZnSe/ZnS quantum dots

6.0 mL of TOA, 220 mg of Zn(Ac)_2_ and 0.42 mL of 1-octanethiol were loaded in a separate three-neck flask^30^. The mixture was heated to 120 °C under N_2_ until a clear solution of Zn(OTT)_2_ precursor was obtained and then heated to 220 °C. The InAs/InP/ZnSe QDs synthesized in the previous step were transferred into the Zn(OTT)_2_ precursor and kept at 280 °C for 1 h. To exchange the surface thiolate ligands with oleate ligands, the reaction solution was cooled to 240 °C, and 5 mL of 0.4 M Zn(OA)_2_ precursors were injected. The reaction was maintained at 240 °C for 1 h. Afterwards, the heating mantle was removed and the reaction mixture was cooled to room temperature. The obtained QDs were precipitated and purified by acetone and toluene and finally redispersed in octane for further QD-LEDs construction.

### Photoluminescence quantum yield measurement

In our experiments, the measurements of absolute PLQY of all QDs samples were conducted using the FLS1000 photoluminescence spectrometer from Edinburgh Instruments, equipped with a near-infrared detector (PMT 1700) spanning the range of 500–1700 nm, and a QYPro™ integrating sphere. All samples were sealed in the same quartz cuvette within an N_2_-filled glovebox. The optical density of the QDs sample was tuned to be ~0.1 at 520 nm. A 520 nm monochromatic exciting light was generated using a 450 W ozone-free xenon arc lamp. During the measurements, the scatter spectra from 500 to 540 nm and PL spectra from 700 to 1200 nm of the reference (toluene) and QDs solution (in toluene) were separately collected with a step size of 0.25 nm. The PLQY values were calculated using the following equation:

1 $${\varphi }_{{\rm{PLQY}}}=\frac{{\int }_{{\lambda }_{3}}^{{\lambda }_{4}}\left|{I}_{{\rm{sample\; Emission}}}-{I}_{{\rm{reference\; Emission}}}\right|{\rm{d}}\lambda }{{\int }_{{\lambda }_{1}}^{{\lambda }_{2}}\left|{I}_{{\rm{reference\; scatter}}}-{I}_{{\rm{sample\; scatter}}}\right|{\rm{d}}\lambda }$$

where *λ*_1_ and *λ*_2_ were the start point and the end point of the fitting range of scatter spectra, respectively; *λ*_3_, and *λ*_4_ were the start point and the end point of the fitting range of emission spectra of QDs samples, respectively. *I* was the intensity of the corresponding excitation or emission wavelength of the reference or the QDs samples.

### Characterizations of quantum dots

The absorption spectrum was measured by a JASCO V-770 spectrophotometer. The steady PL spectra, absolute PLQY and transient PL lifetimes were performed on an Edinburgh Instruments FLS1000 PL spectrometer with a 450 W ozone-free xenon arc lamp. The PLQY was measured by an integrating-sphere accessory and an excitation wavelength of 520 nm. The fluorescence lifetime was measured by time-correlated single photon counting (TCSPC) spectrometer with a picosecond pulsed laser. The XRD measurements were collected on Bruker D8 Advance diffractometer with Cu Kα radiation. The TEM and HRTEM were performed on Thermo Fisher Scientific Talos L120C and Talos F200X G2 microscopes. HAADF-STEM and elemental mapping analysis were performed on a FEI Themis Z. The XPS measurements were carried out on an ESCALAB Xi+ X-ray photoelectron spectrometer (Thermo Fisher) with a monochromatic Al Kα X-ray source.

### Fabrication of quantum dot light-emitting diodes

The ETLs of ZnMgO nanocrystals (NCs) were synthesized through a typical synthetic method^43^. 1.5 mmol of Zn(Ac)_2_ and magnesium acetate (the molar ratio of Mg/Zn, 1/9) were dissolved in 15.0 mL of dimethyl sulfoxide (DMSO). 2.5 mmol of tetramethylammonium hydroxide (TMAH) was dissolved in 5.0 mL of ethanol. The above two solutions were mixed and stirred for 1 h under ambient conditions. ZnMgO NCs were purified and dispersed in ethanol with a concentration of 30 mg mL^−1^. QD-LEDs were fabricated on the glass substrate with pre-patterned ITO (sheet resistance: ~20 Ω sq^−1^). The substrates were ultrasonically cleaned with deionized water, acetone and isopropyl alcohol for 15 min in each step. The substrates were spin-coated with PEDOT:PSS (AI 4083) and baked at 140 °C for 15 min in air. Then, these substrates were transferred to a N_2_-filled glovebox for HTLs deposition. For the standard HTLs, TFB was dissolved in chlorobenzene (8 mg mL^−1^). The TFB solution was then spin-coated with the speed of 3000 rpm, followed by baking at 150 °C for 30 min. For the crosslinked HTLs, a blended solution of TFB and CBPV (total concentration: 8 mg mL^−1^; the weight ratio of TFB/CBPV is between 4/1 and 3/1) was spin-coated (3000 rpm), annealed at 150 °C for 30 min, followed by UV-irradiation (254 nm, 8 W UV-lamp) for 30 min to trigger the crosslinking reaction of CBPV. Subsequently, QDs (20 mg mL^−1^, in n-octane) were spin-coated at 2500 rpm as the emitting layer and baked at 60 °C for 30 min, followed by spin-coating PVP (10 mg mL^−1^) at 2500 rpm and annealing at 100 °C for 20 min. Next, ZnMgO NCs (30 mg mL^−1^) were spin-coated at 3000 rpm as ETL and baked at 60 °C for 30 min. These samples were then loaded into a custom high-vacuum deposition chamber (background pressure, ~3 × 10^−7^ torr) to deposit the top Al cathode (100 nm thick), forming an active device area of 4 mm^2^.

### Characterizations of quantum dot light-emitting diodes

The cross-section images of QD-LEDs were collected by FEI Talos F200X TEM. UV photoelectron spectroscopy (UPS) of HTLs was carried out on a Thermo Scientific ESCALAB 250 XI with a HeI photon source (21.22 eV). Current density-voltage-radiance characteristics for NIR QD-LEDs were analyzed using a computer-controlled Keithley 2400 current/voltage source meter and a Picoammeter (Keithley 6485) with a calibrated Newport silicon diode. The EL spectra were measured using an Ocean Optics spectrometer (USB2000) and a Keithley 2400 source meter. The EQE is calculated according to the following formula by assuming that the emission obeys a Lambertian profile^44,45^

2 $${\eta }_{{\rm{ext}}}=\frac{q{I}_{\det }\int \lambda {\rm{S}}\left(\lambda \right){\rm{d}}{{\lambda }}}{{hcf}\xi \cdot {I}_{{\rm{LEDs}}}}=\frac{q{I}_{\det }\int \lambda {\rm{S}}\left(\lambda \right){\rm{d}}{{\lambda }}}{{hcf}\int {\rm{S}}\left(\lambda \right){\rm{R}}\left(\lambda \right){\rm{d}}{{\lambda }}\cdot {I}_{{\rm{LEDs}}}}=\frac{q\int \lambda {\rm{S}}\left(\lambda \right){\rm{d}}{{\lambda }}}{{hcf}\int {\rm{S}}\left(\lambda \right){\rm{R}}\left(\lambda \right){\rm{d}}{{\lambda }}}\times \frac{{J}_{\det }}{{J}_{{\rm{LEDs}}}}$$

where *q* is the electron charge, *h* is the Planck constant, *c* is the velocity of light, *f* is the fraction of photon reaching photo-detector, S(*λ*) is normalized spectrum of QD-LEDs, and R(*λ*) is the responsivity of photo-detector. *J*_LEDs_ and *J*_det_ are device current density and photo-detector current density, respectively, which are measured from the *J-V* characteristics. $f=\frac{{a}^{2}}{{a}^{2}+{r}^{2}}$, where *r* is the perpendicular distance between the photodetector and the QD-LEDs, and *a* is the radius of the photodetector). The operational stability of QD-LEDs devices was measured using an OLED aging lifetime test system (CRYSCO, D3000-adv).

### Modeling

All first-principles DFT calculations were carried out using projector-augmented wave (PAW) potentials and Perdew-Burke-Ernzerhof (PBE) exchange correlation functional, as implemented in Vienna Ab-Initio Simulation Package (VASP)^46–49^. The kinetic energy cutoff was set at 450 eV. All atomic forces and electronic energies were fully relaxed to less than 10^−2 ^eV Å^−1^ and 10^−5 ^eV, respectively.

For interfacial energy calculations, we considered three low-index facets: the polar {100} and {111} facets, and the nonpolar {110} facet, as identified by XRD patterns of InAs QDs. The InAs surfaces were described by using a slab model consisting of eight InAs monolayers. To avoid spurious effects from the periodic boundary conditions, a vacuum layer greater than 12 Å was added perpendicular to the slab. We employed a symmetric slab model for both {110} and {100} facets^50^, while a wedge model for {111} facets^51,52^. For the calculations of interfacial energy, the cation-rich condition is considered, i.e., the cation chemical potential is chosen as the maximum value, aligning with experimental conditions. We considered a single monolayer of ZnSe epitaxially grown on the InAs substrate to optimize computational efficiency. The resulted interfacial energy *γ* is,

3 $$\gamma={\gamma }_{{\rm{bare}}}+({E}_{{\rm{slab}}\left({\rm{ligand}}\right)}-{E}_{{\rm{slab}}\left({\rm{InAs}}\right)}-{E}_{{\rm{bulk}}\left({\rm{ZnSe}}\right)}-{n}_{{\rm{ligand}}}{\mu }_{{\rm{ligand}}})/A$$

where γ_bare_ is the surface energy of the bare InAs slab, *E*_slab(ligand)_ and *E*_slab(InAs)_ are the total energies of the slab that includes the InAs substrate, ZnSe capping layer and ligands, as well as the energy of the slab with only the InAs substrate, respectively. E_bulk(ZnSe)_ represents the bulk energy of ZnSe. *n*_ligand_ is the number of ligands bonded to the surface, while *μ*_ligand_ is the chemical potential of the ligand. A is the surface area of the slab model.

The binding energy (${E}_{{\rm{b}}}$) of a given chemical specie *A* attached to the slab is defined as follows:

4 $${E}_{{\rm{b}}}={E}_{{\rm{slab}}}+{\mu }_{{\rm{A}}}-{E}_{{\rm{slab}}+{\rm{A}}},$$

where ${E}_{{\rm{slab}}+{\rm{A}}}$ represents the total energy of the slab with the chemical specie *A*, while ${E}_{{\rm{slab}}}$ is the total energy of the slab without *A*. ${\mu }_{{\rm{A}}}$ is the chemical potential of *A*. We note that all binding energies are calculated under optimal ligand coverage.

## Supplementary information

Supplementary Information

Transparent Peer Review file

## Source data

Source Data

## Data Availability

All data that support the findings in this study are available from the corresponding authors upon request. Source data are provided with this paper.
