# Supplementary material for: Efficient and stable near-infrared InAs quantum dot light-emitting diodes
Source: Nat Commun. 2025 Mar 12;16:2450. doi: 10.1038/s41467-025-57746-1 (PMC11897344; doi:10.1038/s41467-025-57746-1)
Supplement: Supplementary file 1 — Supplementary Information [file 41467_2025_57746_MOESM1_ESM.docx]

Supplementary Information for

**Efficient and stable near-infrared InAs quantum dot light-emitting diodes**

Binghan Li^1,2,‡^, Yu Wang^3,‡^, Jiancheng Zhang^1,2,‡^, Yaobo Li^4,‡^, Bo Li^5^, Qingli Lin^3^, Ruijia Sun^1^, Fengjia Fan^5^, Zaiping Zeng^4,*^, Huaibin Shen^3,*^, and Botao Ji^1,2,*^

^1^Zhejiang Key Laboratory of 3D Micro/Nano Fabrication and Characterization, School of Engineering, Westlake University, Hangzhou 310030, China.

^2^Westlake Institute for Optoelectronics, Fuyang, Hangzhou, 311421, China

^3^Key Laboratory for Special Functional Materials of Ministry of Education, National

& Local Joint Engineering Research Center for High-efficiency Display and Lighting

Technology, Henan University, Kaifeng 475004, China.

^4^Henan International Joint Laboratory of Quantum Dot Materials, School of Materials Science and Engineering, Henan University, Kaifeng, Henan 475001, China

^5^CAS Key Laboratory of Microscale Magnetic Resonance and School of Physical Sciences, University of Science and Technology of China, Hefei 230026, China

^‡^These authors contributed equally: Binghan Li, Yu Wang, Jiancheng Zhang, Yaobo Li

^*^E-mail: jibotao@westlake.edu.cn; shenhuaibin@henu.edu.cn; zaiping.zeng@henu.edu.cn

**Supplementary Figures**


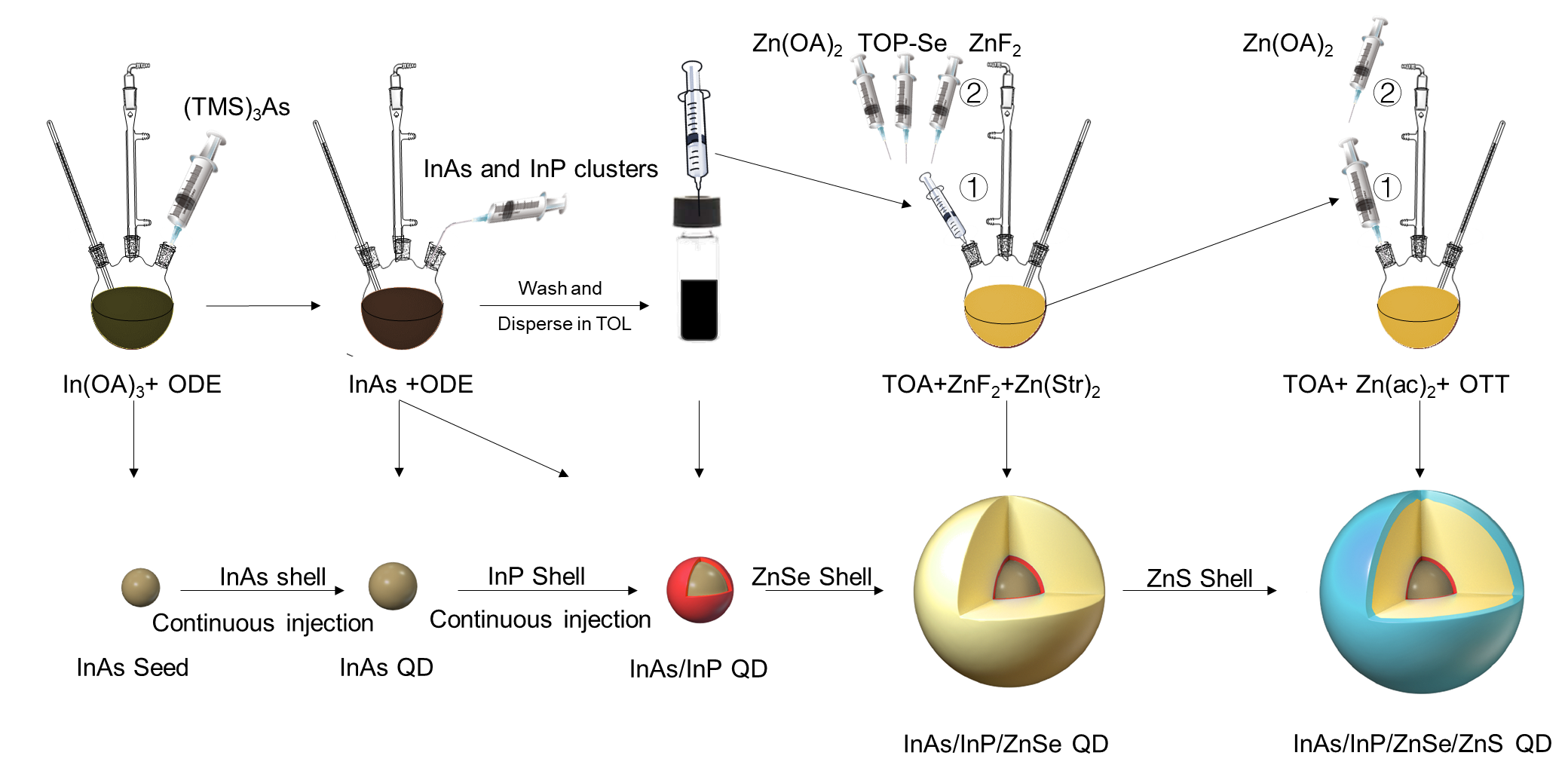


**Supplementary Fig. 1** **|** **Synthesis route of InAs/InP/ZnSe/ZnS QDs.** The scheme shows the synthesis of colloidal large-sized InAs/InP/ZnSe/ZnS core/multishell quantum dots (QDs) with a thick ZnSe shell. Colors represent the InAs (brown-yellow), InP (red), ZnSe (yellow), and ZnS (sky blue).


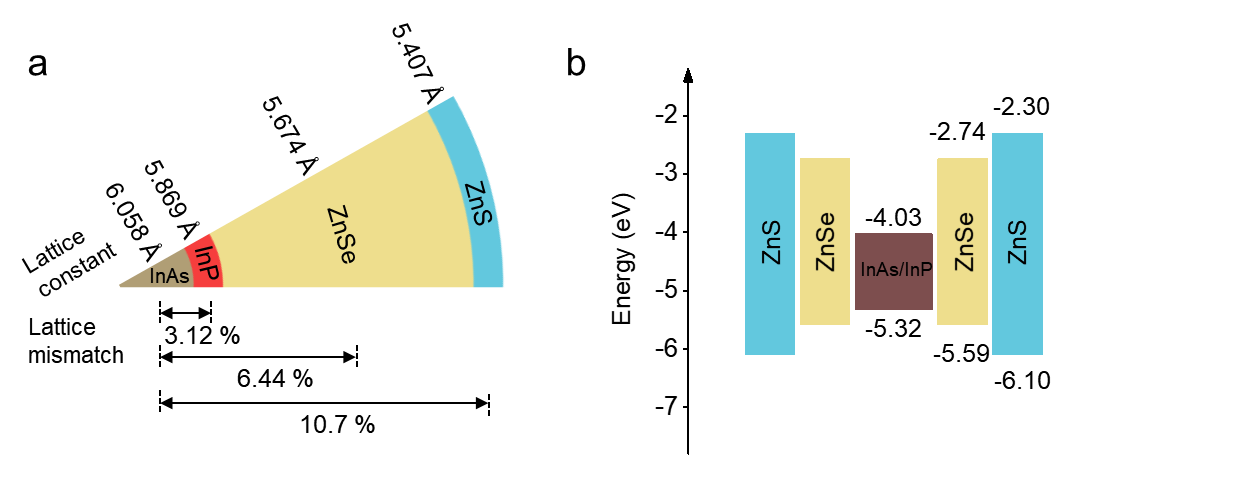


**Supplementary Fig. 2** **|** **Lattice mismatch and band alignment in QDs. a** Lattice parameters and lattice mismatch between bulk cubic InAs and bulk cubic shell materials. **b** Energy level diagram of InAs/InP/ZnSe/ZnS QDs, with the E_VB_/E_CB_ of bulk ZnSe and ZnS adopted from Supplementary Ref. 1.


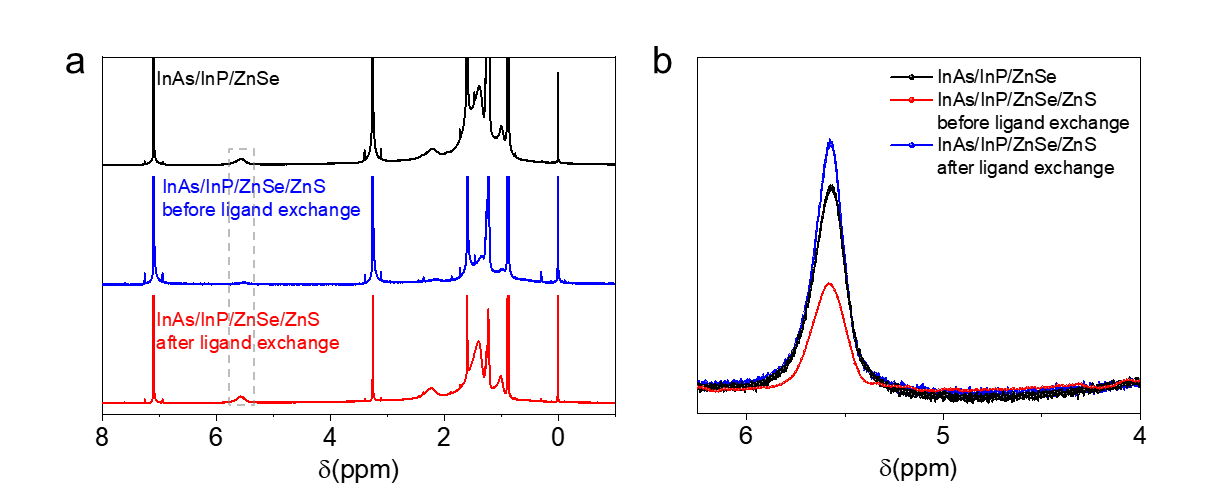


**Supplementary Fig. 3** **|** **^1^H NMR analysis of ligand exchange of QDs. a** ^1^H NMR spectra of InAs/InP/ZnSe QDs (black), InAs/InP/ZnSe/ZnS QDs before (blue) and after (red) ligand exchange. All NMR spectra were normalized according to the concentration of QDs and the signal intensity of tetramethylsilane (internal standard substance) at around 0 ppm. **b** Zoom on the alkene resonance (~5.7 ppm) in ^1^H NMR spectra in (**a**).


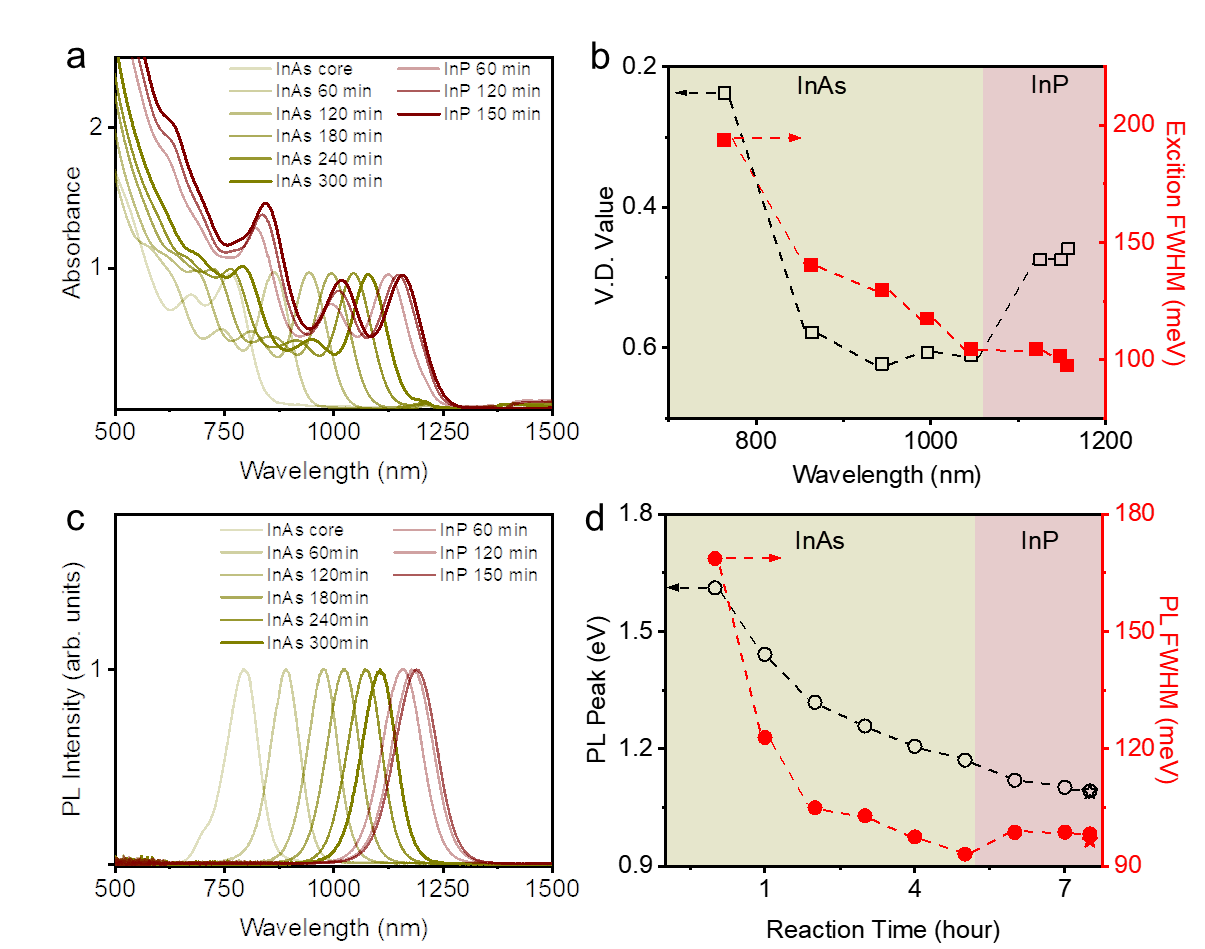


**Supplementary Fig. 4** **| Evolution of absorption and PL spectra during InAs/InP QDs (~1160 nm) synthesis.** Temporal evolution of (**a**) absorption spectra and (**c**) PL spectra recorded during the synthesis of InAs/InP core/shell QDs with an exciton peak at ~1160 nm starting from small InAs seeds. **b** The evolution of V.D. values (hollow black square) and the full widths at half-maximum (FWHMs) of the first excitonic absorption peaks (solid red square) during the synthesis of the sample. The InAs and InAs/InP QDs are marked by the light brown-yellow area and the light pink area, respectively. The valley depth $(V.D.)$ values are calculated by measuring the first excitonic absorption peak and valley value: $V.D. =1-({Abs}_{\mathrm{valley}}/{Abs}_{\mathrm{peak}})$. The FWHMs of the first excitonic absorption peaks are obtained by fitting the rising band edge of the corresponding absorption spectra using a Gaussian function. **d** The evolution of emission peaks (hollow black circle) and emission FWHMs (solid red circle) during the synthesis of the sample as a function of reaction time. The InAs and InAs/InP QDs are marked by light brown-yellow area and light pink area, respectively. The five-pointed star represents the purified InAs/InP QDs used for the following growth of ZnSe shell.


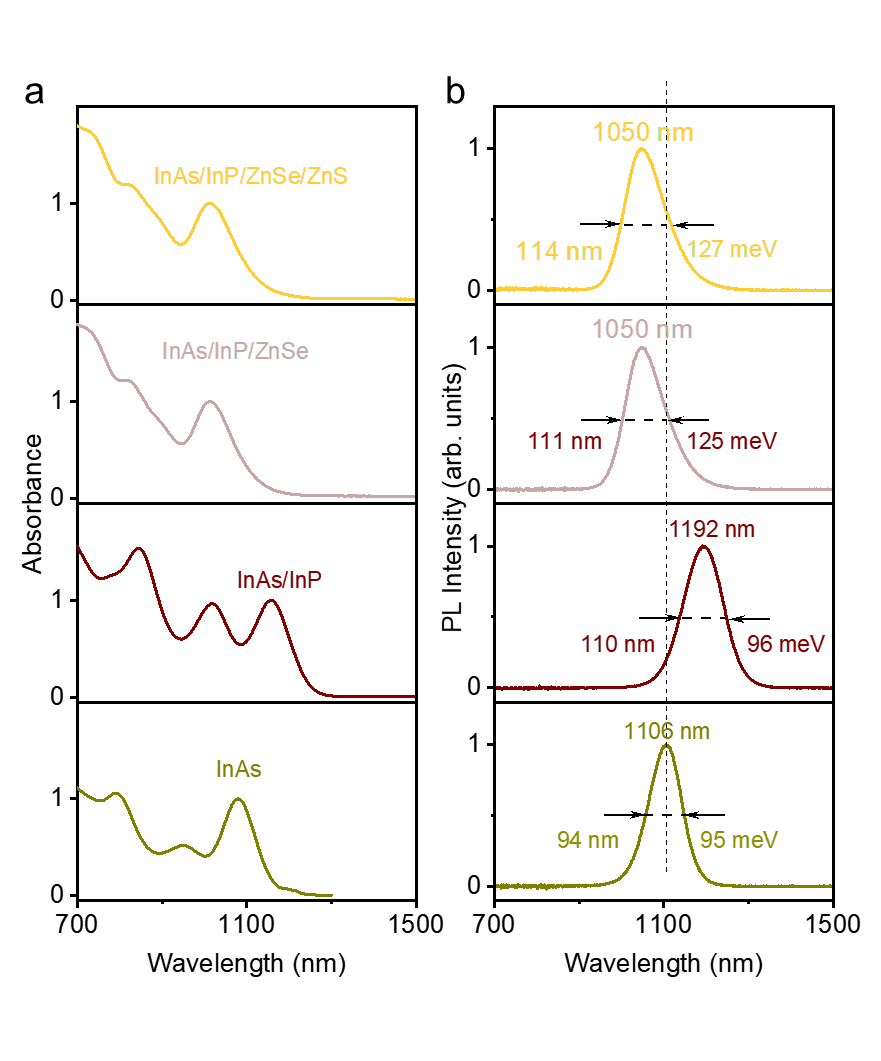


**Supplementary Fig. 5 | Evolution of absorption and PL spectra during InAs/InP/ZnSe/ZnS QDs (~1050 nm) synthesis.** Evolution of (**a**) absorption spectra and (**b**) PL spectra of large-sized InAs/InP/ZnSe/ZnS QDs with an emission peak at ~1050 nm. The emission wavelength of InAs QDs (brown-yellow) was located at ~1106 nm and then shifted to ~1192 nm upon the shell growth of InP (dark red). Subsequently, multiple ZnSe (light red) and ZnS (yellow) layers were grown on InAs/InP core/shell QDs through the developed synthetic procedure. This produces InAs/InP/ZnSe/ZnS QDs emitting at ~1050 nm in the short-wavelength infrared range with a narrow ensemble PL linewidth of 127 meV. All of the samples were washed and redispersed in tetrachloroethylene prior to the spectra measurements.


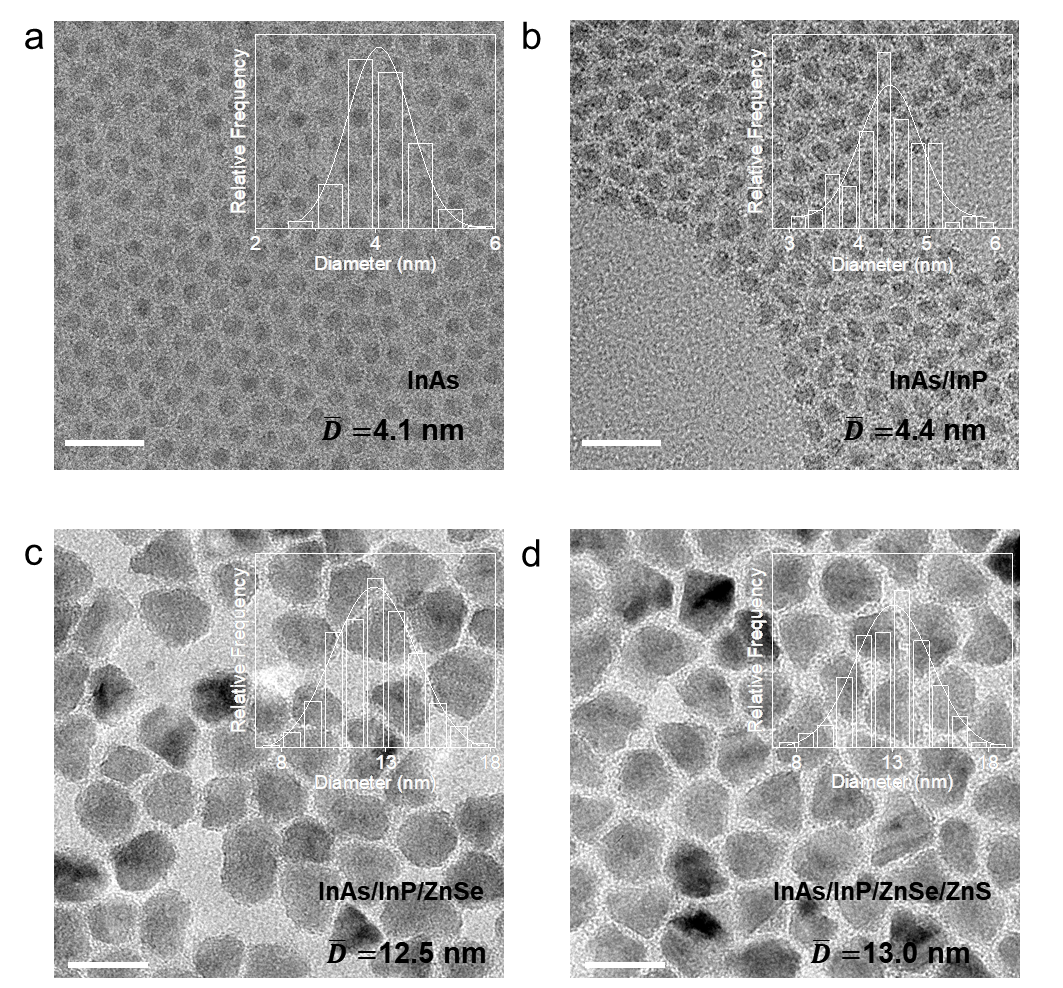


**Supplementary Fig. 6** **|** **Size evolution of InAs/InP/ZnSe/ZnS QDs (~1050 nm).** TEM images of (**a**) InAs, (**b**) InAs/InP, (**c**) InAs/InP/ZnSe and (**d**) InAs/InP/ZnSe/ZnS QDs with a final PL peak at ~1050 nm. All the scale bars are equal to 20 nm. Corresponding size distribution histograms and average diameters are also indicated in the TEM images.


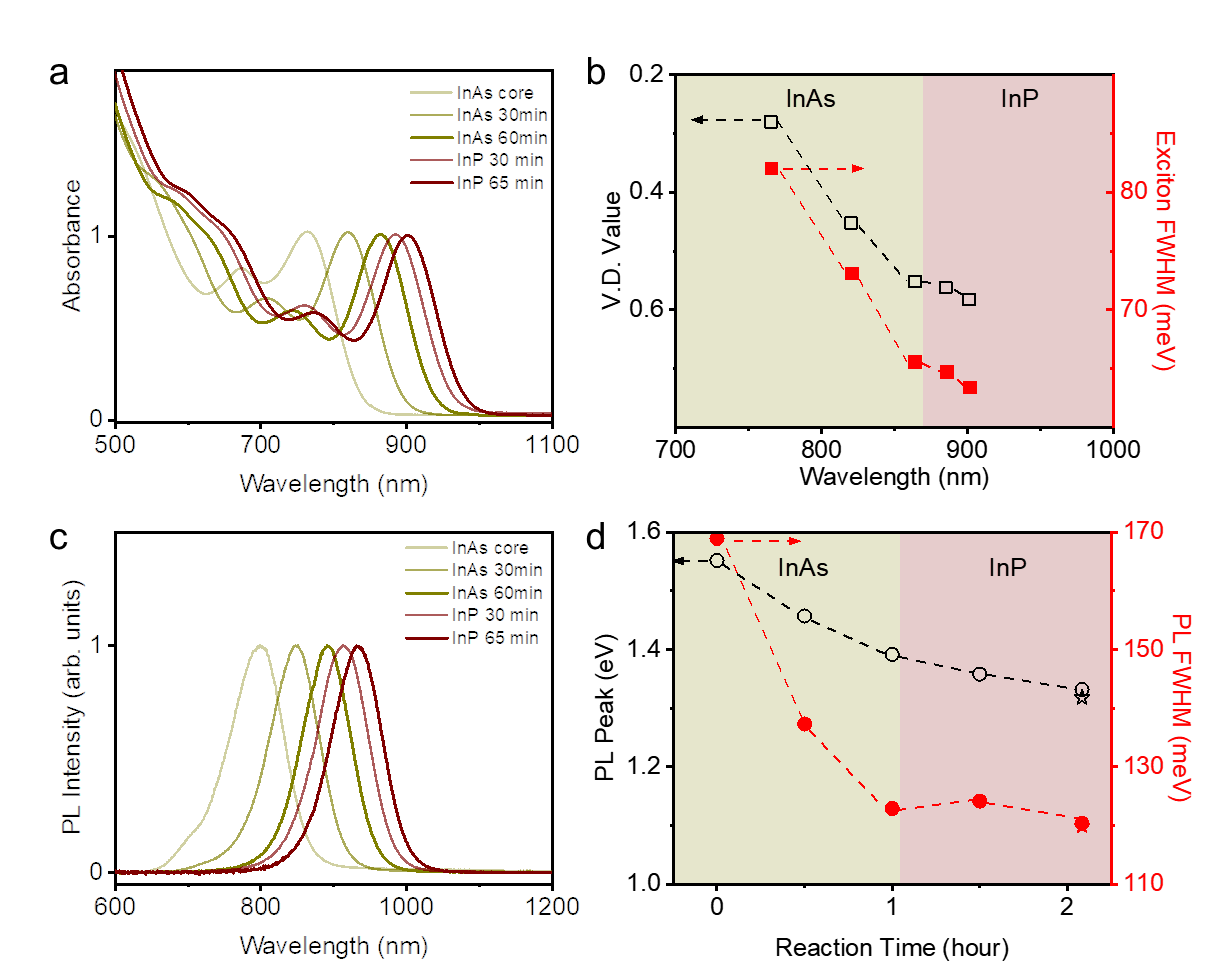


**Supplementary Fig. 7** **| Evolution of absorption and PL spectra during the synthesis of InAs/InP QDs for QDs-900. a,c** Evolution of (**a**) absorption spectra and (**c**) PL spectra recorded during the synthesis process of InAs/InP core/shell QDs (for QDs-900) starting from InAs seeds. **b** The evolution of V.D. values (hollow back square) and the FWHMs of the first excitonic absorption peaks (solid red square) during the synthesis of the sample. The InAs and InAs/InP QDs are marked by the light brown-yellow area and the light pink area, respectively. **d** The evolution of emission peaks (hollow back circle) and emission FWHMs (solid red circle) during the synthesis of the sample as a function of reaction time. The InAs and InAs/InP QDs are marked by the light brown-yellow area and the light pink area, respectively. The purified InAs/InP QDs used for the subsequent growth of ZnSe and ZnS shells are indicated by five-pointed stars. All of the absorption and emission spectra were measured by dispersing the QDs in tetrachloroethylene.


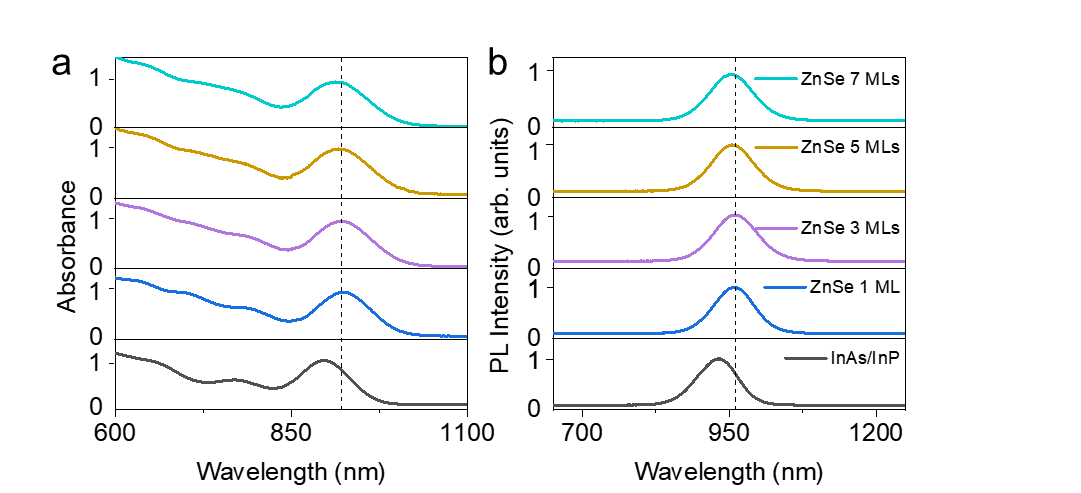


**Supplementary Fig. 8** **| Evolution of absorption and PL spectra during the synthesis of InAs/InP/ZnSe QDs for QDs-900. a,b** Evolution of (**a**) absorption spectra and PL spectra of InAs/InP/ZnSe QDs with increasing thickness of the ZnSe shell (1 monolayer (ML) to 7 MLs) during the synthesis of QDs-900. Colors represent the InAs/InP (black), ZnSe 1ML (blue), ZnSe 3 MLs (purple), ZnSe 5 MLs (yellow), and ZnSe 7 MLs (sky blue).


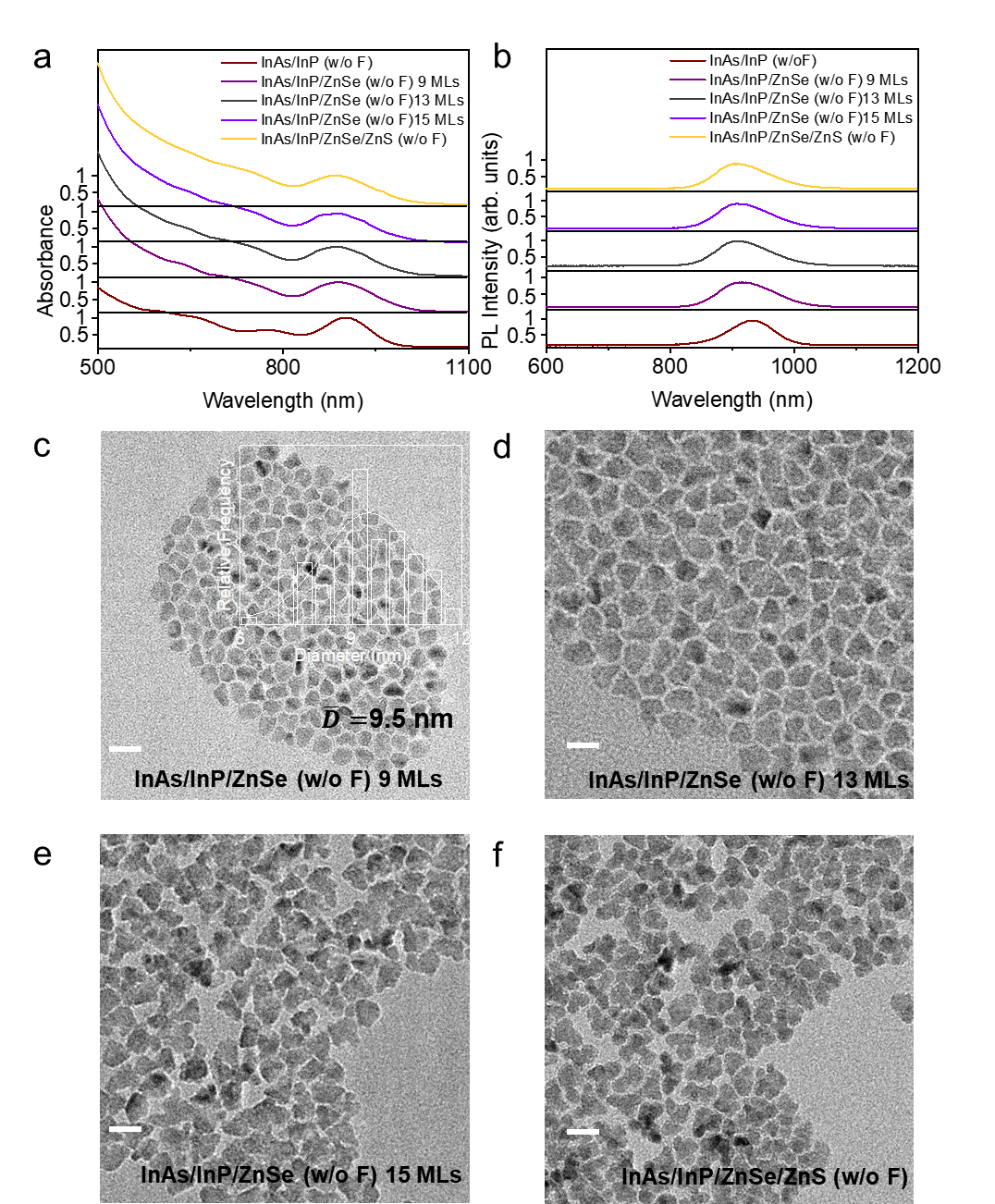


**Supplementary Fig. 9** **|** **Spectroscopic and morphological evolution during the synthesis of InAs/InP/ZnSe/ZnS QDs without ZnF_2_.** **a**,**b** Evolution of (**a**) absorption spectra and (**b**) PL spectra of large-sized InAs/InP/ZnSe/ZnS core/multishell QDs (emission peak, ~900 nm) without the utilization of ZnF_2_ (w/o F) while maintaining other conditions unchanged. Colors represent the InAs/InP (w/o F) (red), InAs/InP/ZnSe (w/o F) 9 monolayers (MLs) (purple), InAs/InP/ZnSe (w/o F) 13 MLs (gray), InAs/InP/ZnSe (w/o F) 15 MLs (violet) and InAs/InP/ZnSe/ZnS (w/o F) (yellow). **c**–**f** TEM images of (**c**–**e**) InAs/InP/ZnSe QDs with increasing thickness of the ZnSe shell (9 to 15 MLs) and (**f**) InAs/InP/ZnSe/ZnS QDs synthesized in the absence of ZnF_2_ additive. The thicknesses of the ZnSe shell are marked in the TEM images in (**c**–**e**). Corresponding size distribution histograms and average diameters are inserted in the TEM images. All the scale bars are equal to 20 nm.


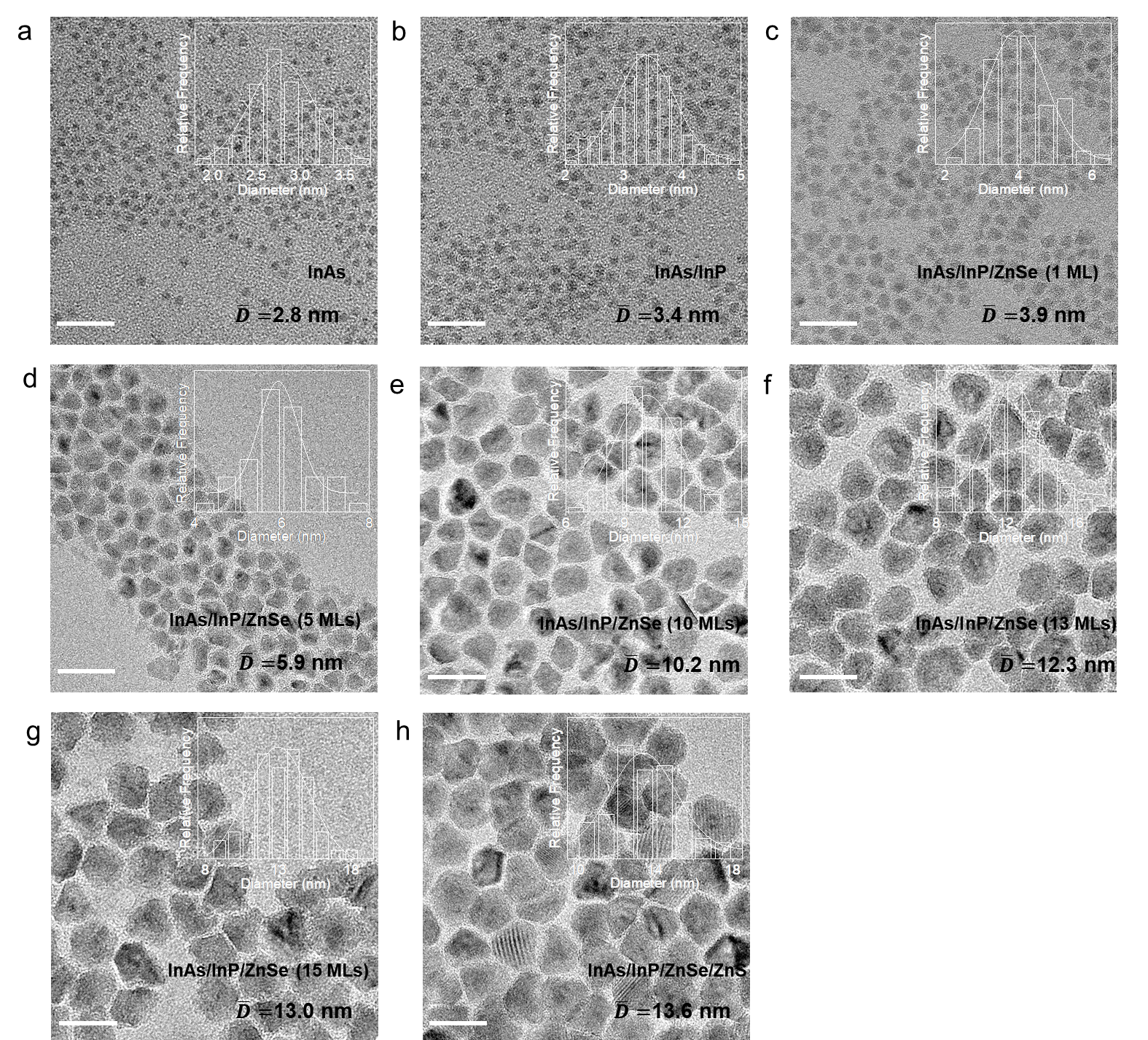


**Supplementary Fig. 10** **| Morphological evolution of QDs-900.** TEM images of (**a**) InAs, (**b**) InAs/InP, (**c**–**g**) InAs/InP/ZnSe with increasing thickness of the ZnSe shell (1 to 15 MLs) and (**h**) InAs/InP/ZnSe/ZnS QDs (QDs-900). Corresponding size distribution histograms and average diameters are also indicated. All the scale bars are equal to 20 nm.


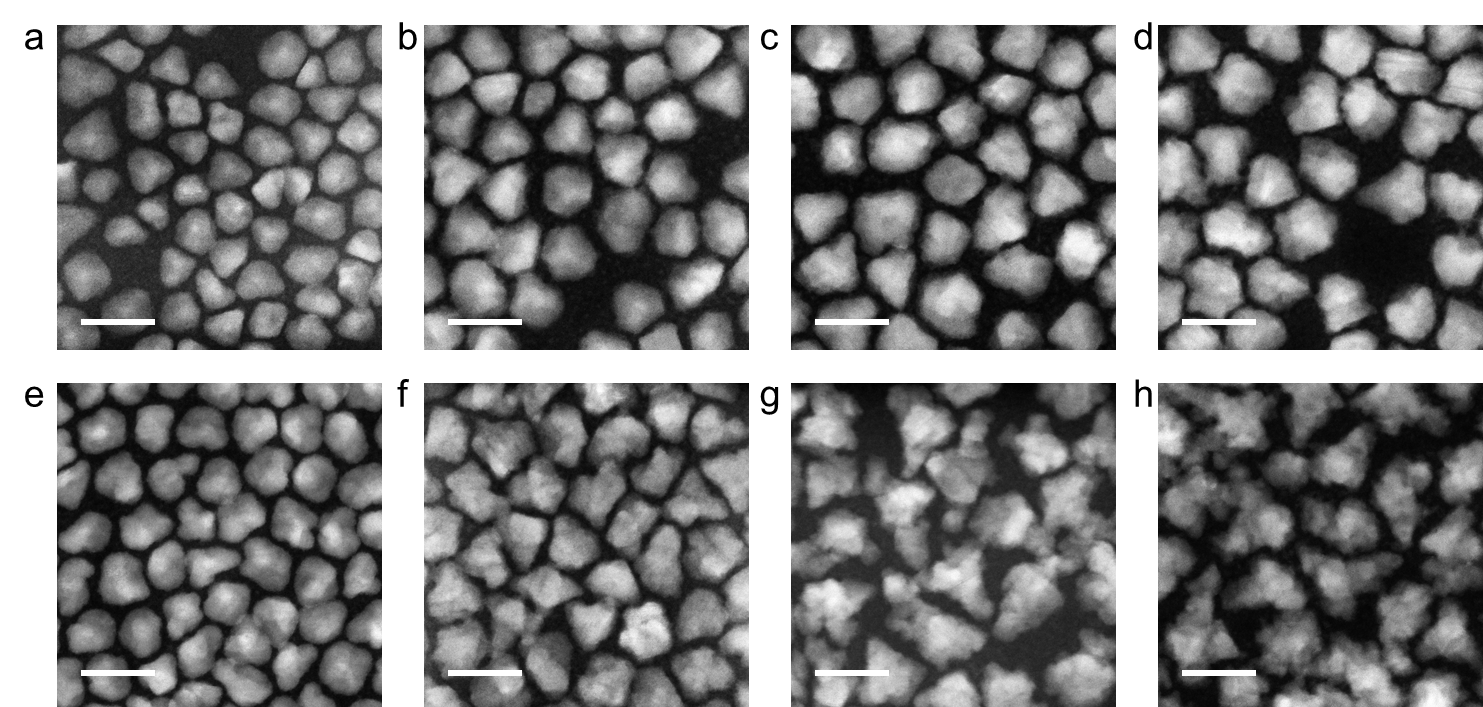


**Supplementary Fig. 11** **| Morphological evolution of QDs-900. a**–**d** STEM images of QDs synthesized with ZnF_2_. InAs/InP/ZnSe with increasing thickness of the ZnSe shell (10MLs **(a)**, 13 MLs **(b)**, 15 MLs **(c)**) and **(d)** InAs/InP/ZnSe/ZnS QDs (QDs-900). **e**–**f** STEM images of QDs synthesized without ZnF_2_. InAs/InP/ZnSe with increasing thickness of the ZnSe shell 10MLs **(e)**, 13 MLs **(f)**, 15 MLs **(g)**) and **(h)** InAs/InP/ZnSe/ZnS QDs. All the scale bars are equal to 20 nm.

**Supplementary Fig. 12** **|** **XRD evolution of QDs-900.** X-ray diffraction patterns (XRD) of InAs (brown), InAs/InP (red), InAs/InP/ZnSe (yellow) and InAs/InP/ZnSe/ZnS QDs (sky blue, QDs-900). The standard XRD patterns of bulk zincblende InAs (Magenta), InP (grey), ZnSe (purple) and ZnS (blue) are also provided for comparison. XRD measurements reveal the original zinc-blende crystalline structure of the InAs cores is inherited, while all diffraction peaks gradually shifted towards higher angles with the outer multishell growth, indicating epitaxial shell deposition.


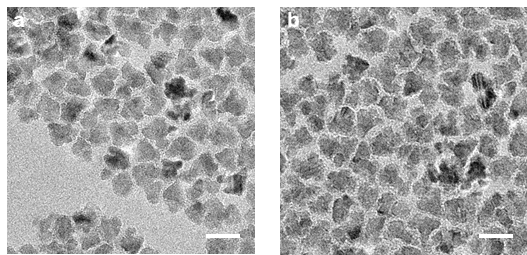


**Supplementary Fig. 13** **|** **Impact of hydrofluoric acid on the morphology of** **InAs/InP/ZnSe/ZnS QDs. a** TEM image of InAs/InP/ZnSe QDs. **b** TEM image of InAs/InP/ZnSe/ZnS QDs. During the synthesis, hydrofluoric acid was introduced to produce oxide-free InAs/InP QDs, on which the ZnSe shell was grown at 340 °C. The amounts of ZnSe and ZnS precursors used are consistent with the standard synthesis outlined in Fig. 1 of the main text. The resulting QDs displayed highly irregular morphology. All the scale bars are equal to 20 nm.


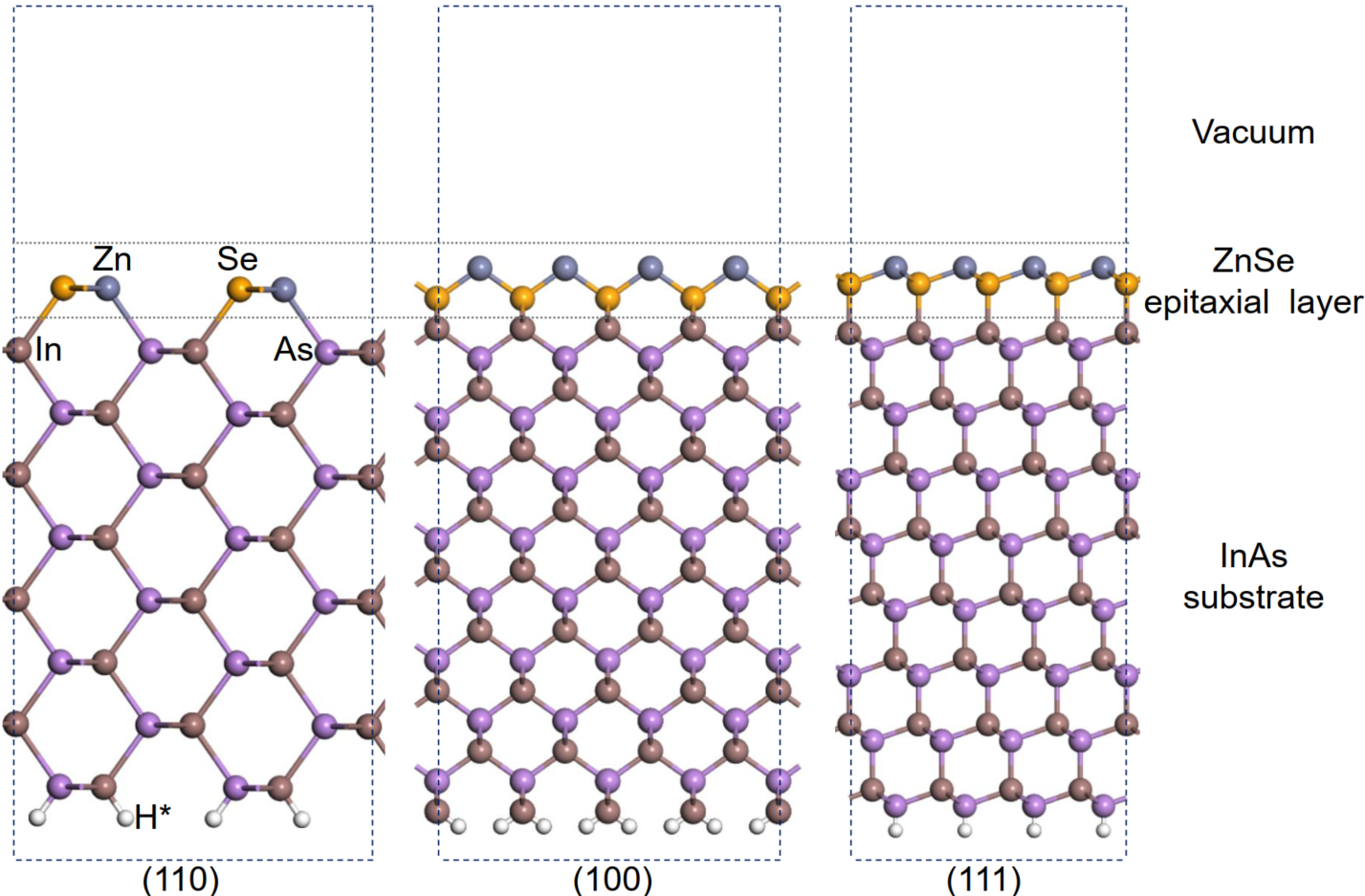


**Supplementary Fig. 14** **|** **Slab model of QD facets with ZnSe epitaxial layer.** Slab model for the {110}, {100}, and {111} facets of QDs, consisting of an InAs substrate with a monolayer ZnSe epitaxial layer. A vacuum layer of ~15 angstroms is included to minimize spurious interactions between periodic images. The dangling bonds on the lower surface are passivated with pseudo-hydrogens (H*) carrying fractional charges of 1.5 e and 0.5 e to passivate the surface cations and anions, respectively. The grey, yellow, brown, purple and white spheres represent the elements Zn, Se, In, As and H, respectively.


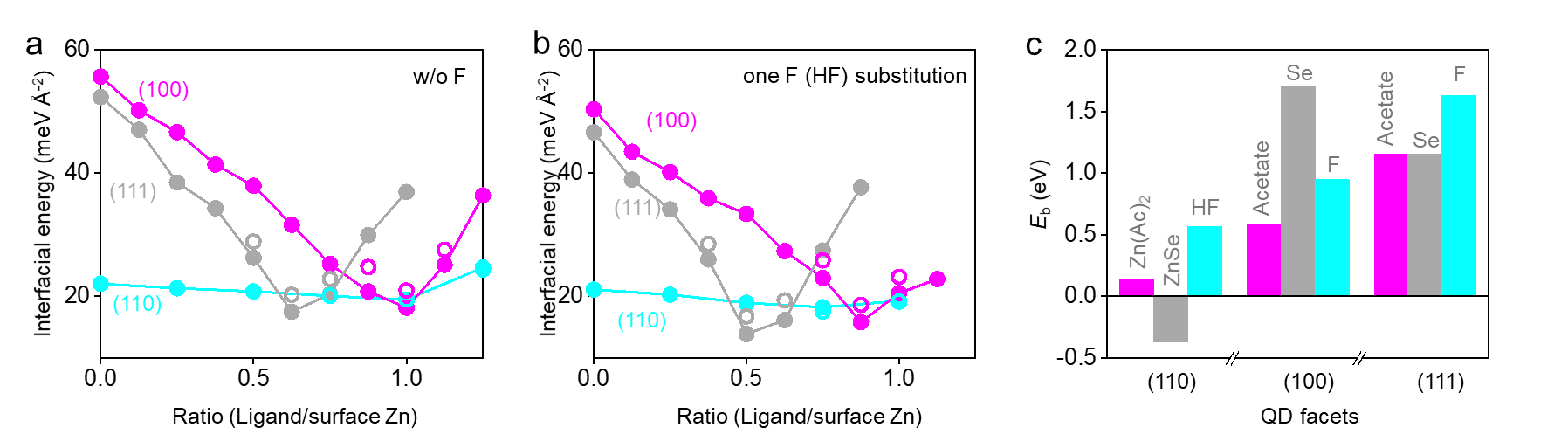


**Supplementary Fig. 15 |** **Effect of InP interlayer between InAs and ZnSe on calculated interfacial and binding energies. a**,**b** Calculated interfacial energies as a function of ligand per surface Zn for the {110} (cyan), {100} (violet), and {111} (gray) facets using a slab model consisting of an InAs substrate with a single epitaxial ZnSe monolayer, shown (**a**) without fluoride species and (**b**) with one original ligand replaced by one F or HF. In both (**a**) and (**b**), open symbols represent results obtained with a monolayer InP bridging layer between the InAs substrate and ZnSe epitaxial layer. This inclusion of the InP layer slightly affects the interfacial energies but does not impact the optimal ligand coverage. **c** Binding energies of various species on the {100}, {111}, and {110} facets at optimal ligand coverage, considering the InP bridging layer in our slab model. The results indicate that the presence of the InP layer does not qualitatively affect the trend of binding energies for different chemical species.


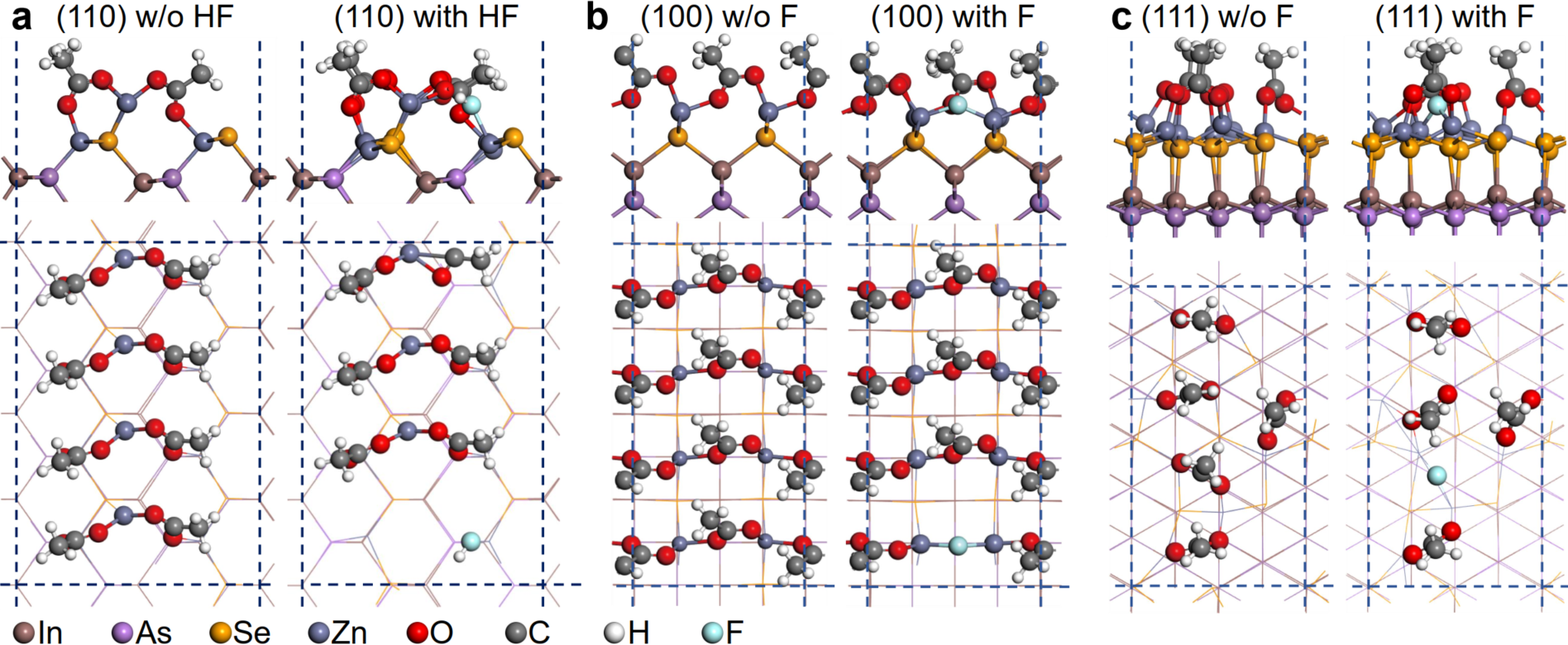


**Supplementary Fig. 16** **|** **Ligand Binding Configurations on QD Facets without and with fluoride species.** Top and side views of ligand binding configuration under the optimal ligand coverage for (**a**) the {110} facet, (**b**) the {100} facet and (**c**) the {111} facet, respectively. These configurations are presented without fluoride species (left) and with one original ligand replaced by one F or HF (right). The grey, yellow, brown, purple, white, red, black and cyan spheres represent the elements Zn, Se, In, As, H, O, C and F, respectively.


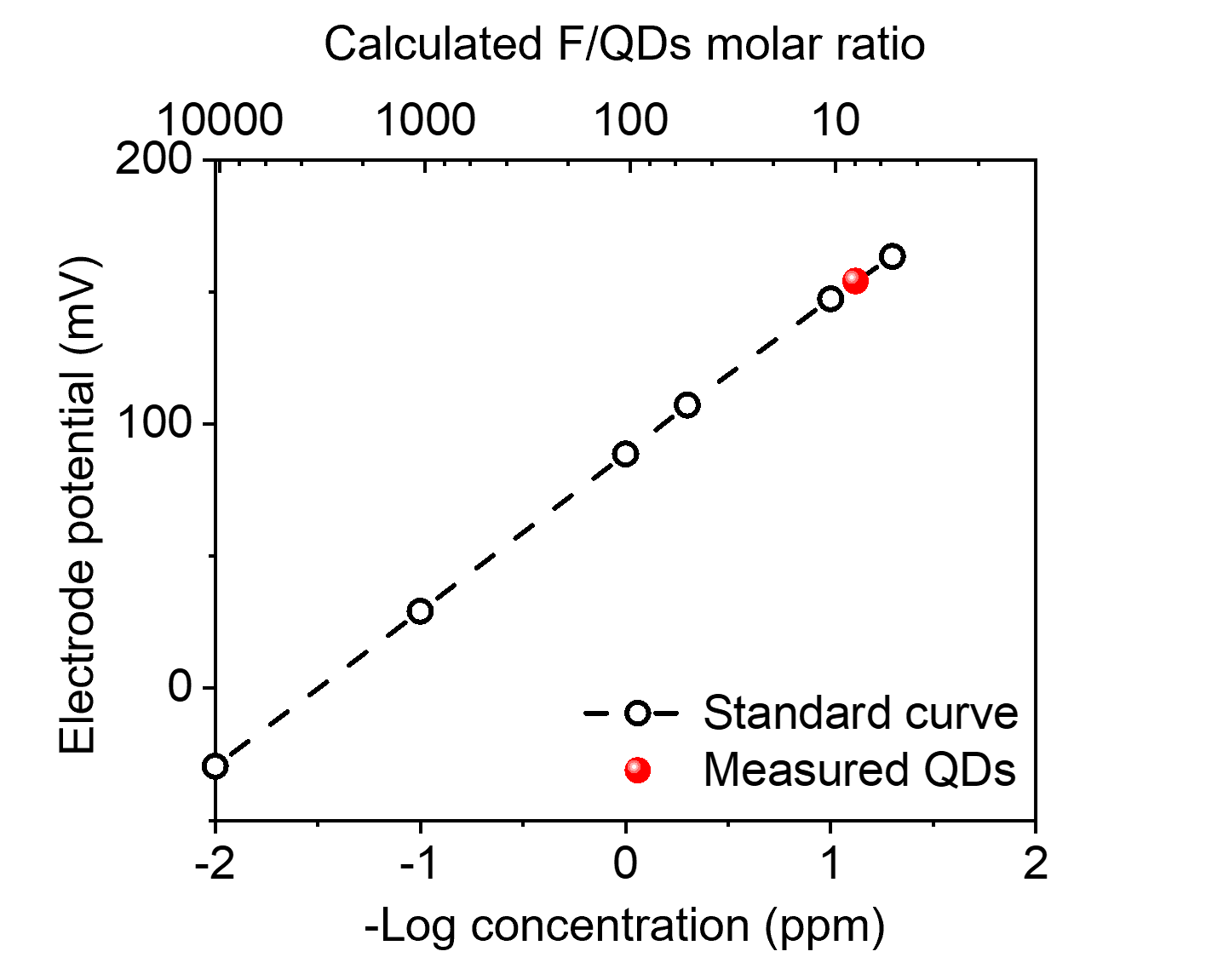


**Supplementary Fig. 17** **|** **Quantification of surface fluoride ions in ZnF_2_-treated QDs.** Quantification of F^-^ ions per QD was performed using a potentiometric method. Approximately 10 nmol of ZnF_2_-treated QDs were thoroughly purified and decomposed with nitric acid in a sealed vial. The concentration of F^-^ ions was determined using a fluoride ion-selective electrode connected to a digital pH meter. A calibration curve (dash line) was established by measuring a series of standard solutions with known F^-^ concentrations (black circle). The analysis revealed a molar ratio of F^-^ ions to QDs of only 8:1 (red ball). This low ratio suggests that the F^-^ ions were not incorporated into the QD lattice but were instead primarily located on the QD surface.


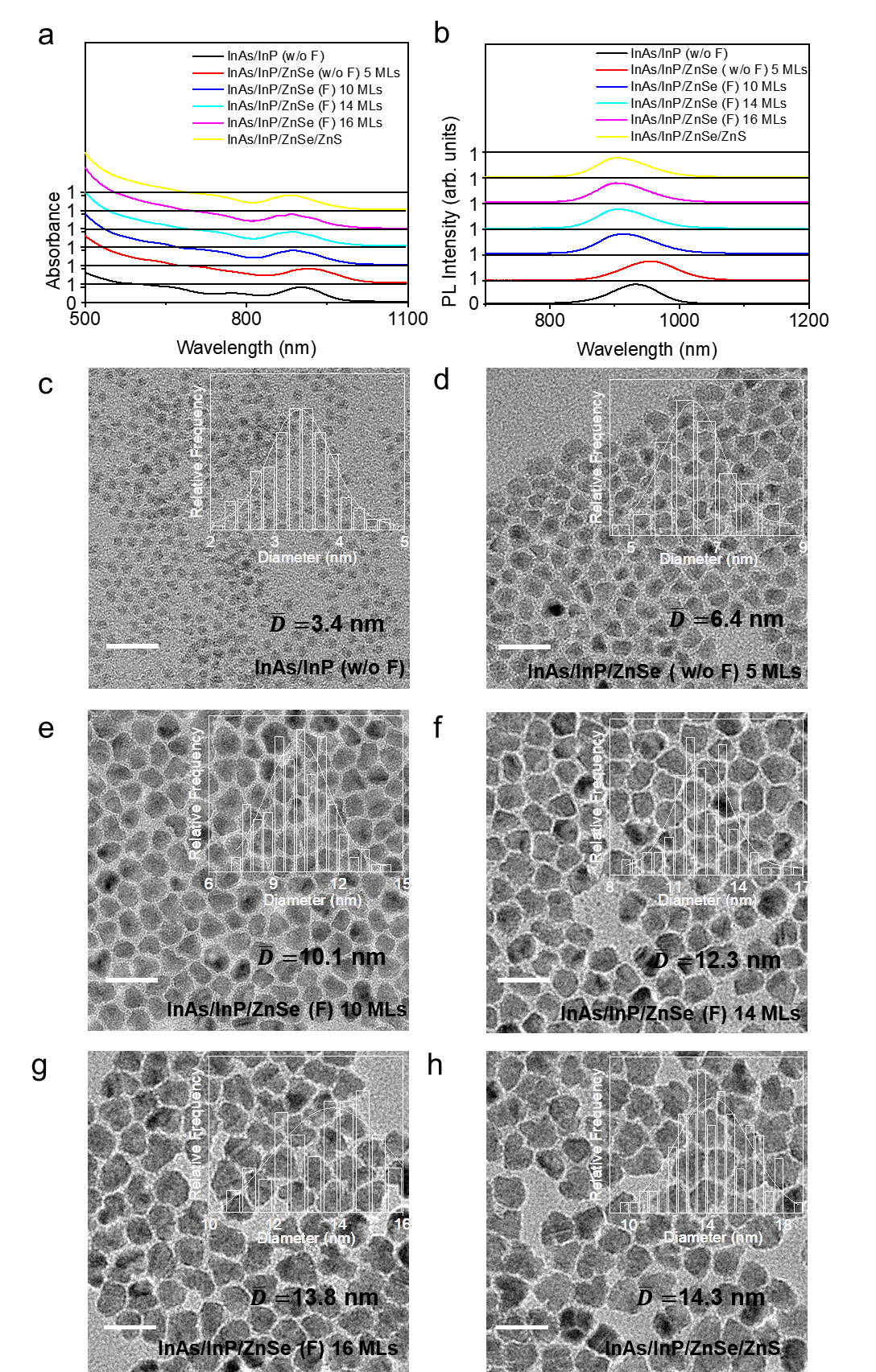


**Supplementary Fig. 18** **|** **Effect of supplementary ZnF_2_ addition on ZnSe shell growth and QD properties.** No inorganic fluoride additives of InF_3_ or ZnF_2_ (w/o F) were used during the synthesis of InAs/InP/ZnSe QDs with 5 MLs ZnSe. ZnF_2_ was then introduced after the shell growth of 5 monolayers (MLs) ZnSe. **a**,**b** Evolution of (**a**) absorption spectra and (**b**) PL spectra of InAs/InP/ZnSe/ZnS QDs (emission peak, ~900 nm). **c**–**h** TEM images of (**c**) InAs/InP (w/o F), **(d)** InAs/InP/ZnSe (w/o F; ZnSe: 5 MLs), (**e**) InAs/InP/ZnSe (with F; ZnSe: 10 MLs), (**f**) InAs/InP/ZnSe (with F; ZnSe: 14 MLs), (**g**) InAs/InP/ZnSe (with F; ZnSe: 16 MLs) and (**h**) InAs/InP/ZnSe/ZnS QDs. Corresponding size distribution histograms and average diameters are also indicated. All the scale bars are equal to 20 nm.


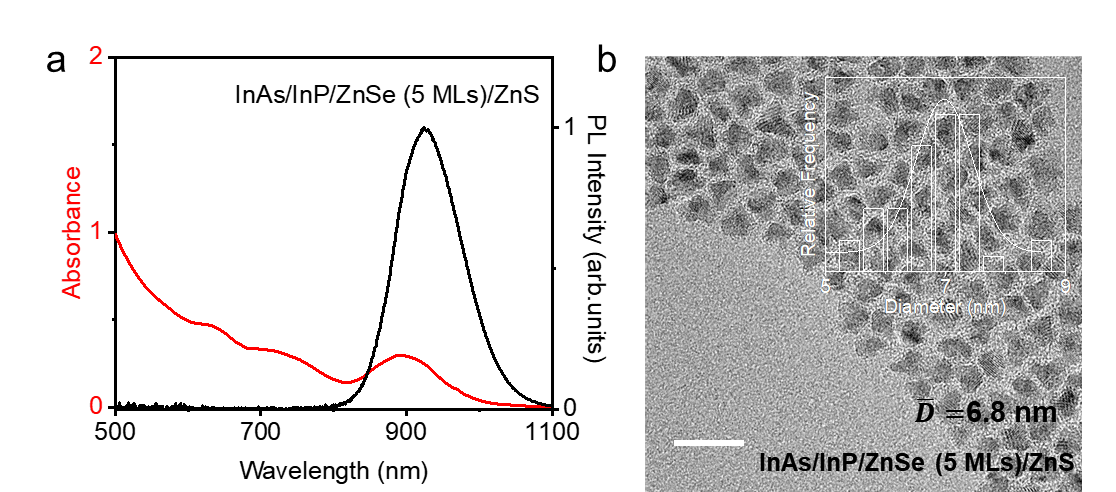


**Supplementary Fig. 19** **|** **Optical properties and morphology of InAs/InP/ZnSe (5MLs)/ZnS QDs. a** Absorption (red) and PL (black) spectra of InAs/InP/ZnSe/ZnS QDs with a 5 monolayers (MLs) ZnSe shell. **b** TEM image of InAs/InP/ZnSe (5 MLs)/ZnS QDs. The corresponding size distribution histogram is indicated in the TEM image. The average diameter of InAs/InP/ZnSe (5 MLs)/ZnS QDs is ~6.8 nm. The scale bar is equal to 20 nm.


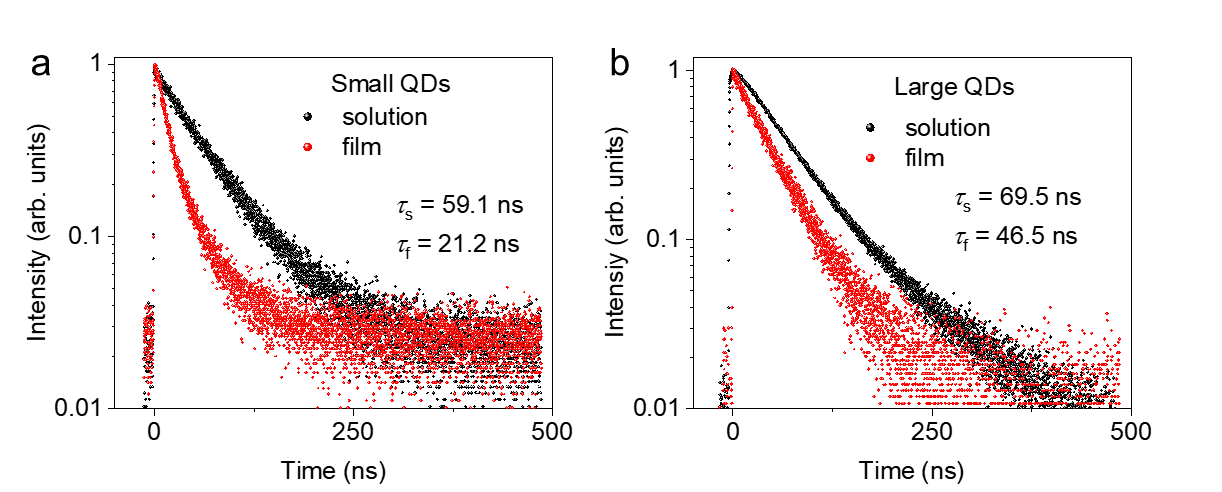


**Supplementary Fig. 20** **|** **Effect of QD size on FRET efficiency.** **a** Time-resolved PL decay curves of small ZnF_2_-treated QDs (6.8 nm) in toluene (black) and deposited on glass as a film (red). **b** Time-resolved PL decay curves of large ZnF_2_-treated QDs (13.0 nm, QDs-900) in toluene (black) and deposited on glass as film (red). The QDs films were encapsulated with glass slides using ultraviolet curing adhesive under an N_2_ atmosphere. The red plot in (**a**) was fitted by a biexponential function. The rest of the PL decays were fitted using monoexponential decay kinetics. Corresponding PL lifetimes are shown in the figures. Note that the PL decay curves of QDs re-dissolved in toluene from QDs films were identical to those of QDs initially dispersed in toluene, regardless of QDs size. This result indicates that defects are minimally formed during the film formation. Therefore, the Förster resonance energy transfer (FRET) efficiency of the QDs film can be calculated by the equation: $\eta_{\mathrm{FRET}}\text{= 1-}\frac{\text{τ}_{\text{f}}}{\text{τ}_{\text{s}}}$, where$\text{ }\text{τ}_{\text{s}}$ is the PL lifetime in solution and$\text{ }\text{τ}_{\text{f}}$ is the lifetime of QDs film. The FRET efficiencies of the small QDs film and the large QDs film are calculated to be 64.1% and 33.1%, respectively, implying the FRET is significantly restrained for large QDs.


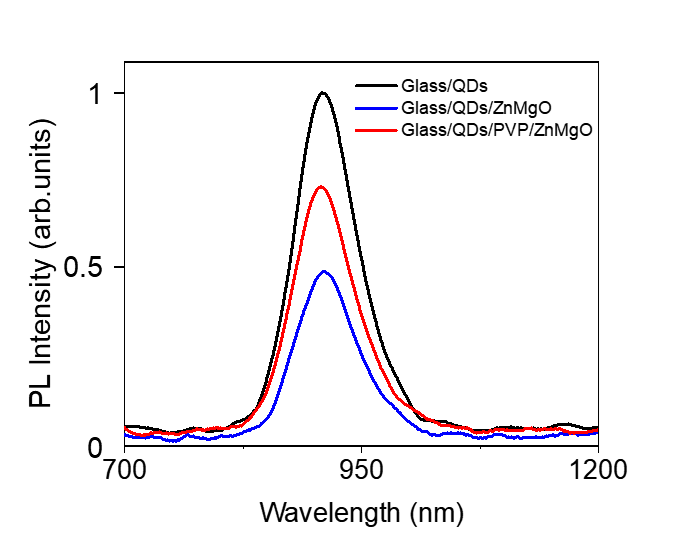


**Supplementary Fig. 21 | PL spectra of QD films with different layer configurations.** The QDs were deposited using the same spin-coating condition as the QD-LEDs fabrication. Black: glass/QDs; blue: glass/QDs/ZnMgO; red: glass/QDs/PVP/ZnMgO. The ultrathin insulating layer of polyvinylpyrrolidone (PVP) inserted between the QDs layer and the ZnMgO layer clearly alleviates the PL quenching of QDs film induced by ZnMgO.

**Supplementary Fig. 22** **|** **Time-resolved PL decays of QDs-900 films on glass versus TFB.** Glass: black; TFB (poly(9,9-dioctylfluorene-co-N-(4-(3-methylpropyl))-diphenylamine): blue. The QDs films were encapsulated with glass slides by ultraviolet curing adhesive under N_2_ atmosphere.


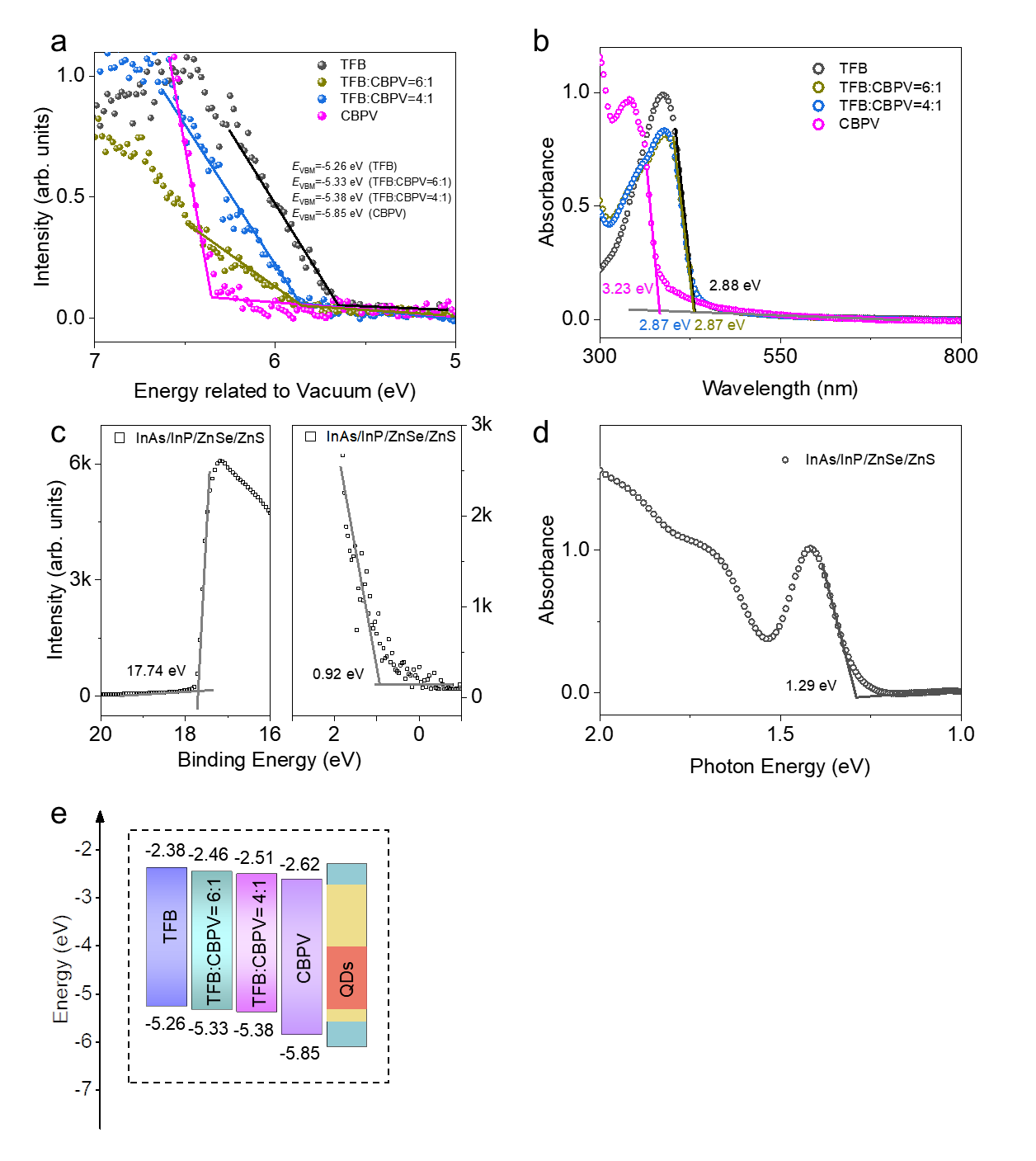


**Supplementary Fig. 23** **|** **Measurement of HOMO/LUMO energy levels of different functional layers and band structure of QDs. a** The highest occupied molecular orbital (HOMO) energies of TFB (poly(9,9-dioctylfluorene-co-N-(4-(3-methylpropyl))-diphenylamine, black), CBPV (4,4′-bis(3-vinyl-9H-carbazol-9-yl)-1,1′-biphenyl, magenta) and TFB:CBPV (6/1 weight ratio of TFB/CBPV, brown-yellow; and 4/1, blue) measured by ultraviolet photoelectron spectroscopy (UPS). **b** Absorption spectra of TFB (black), CBPV (magenta) , TFB:CBPV(6:1, brown-yellow) and TFB:CBPV(4:1, blue). **c,d** The UPS (**c**) and absorption spectra (**d**) of QDs layer (QDs-900). **e** The *E*_VBM(HOMO)_/*E*_CBM(LUMO)_ of TFB (blue), TFB:CBPV (6:1) (light cyan), TFB:CBPV (4:1) (magenta) and CBPV (purple) are calculated to be -5.26/-2.38 eV, -5.33/-2.46 eV, -5.38/-2.51 eV and -5.85/-2.62 eV. The *E*_VB_/*E*_CB_ of InAs/InP core QDs (red), bulk ZnSe (yellow) and ZnS (sky blue) are also shown in (**e**).

**
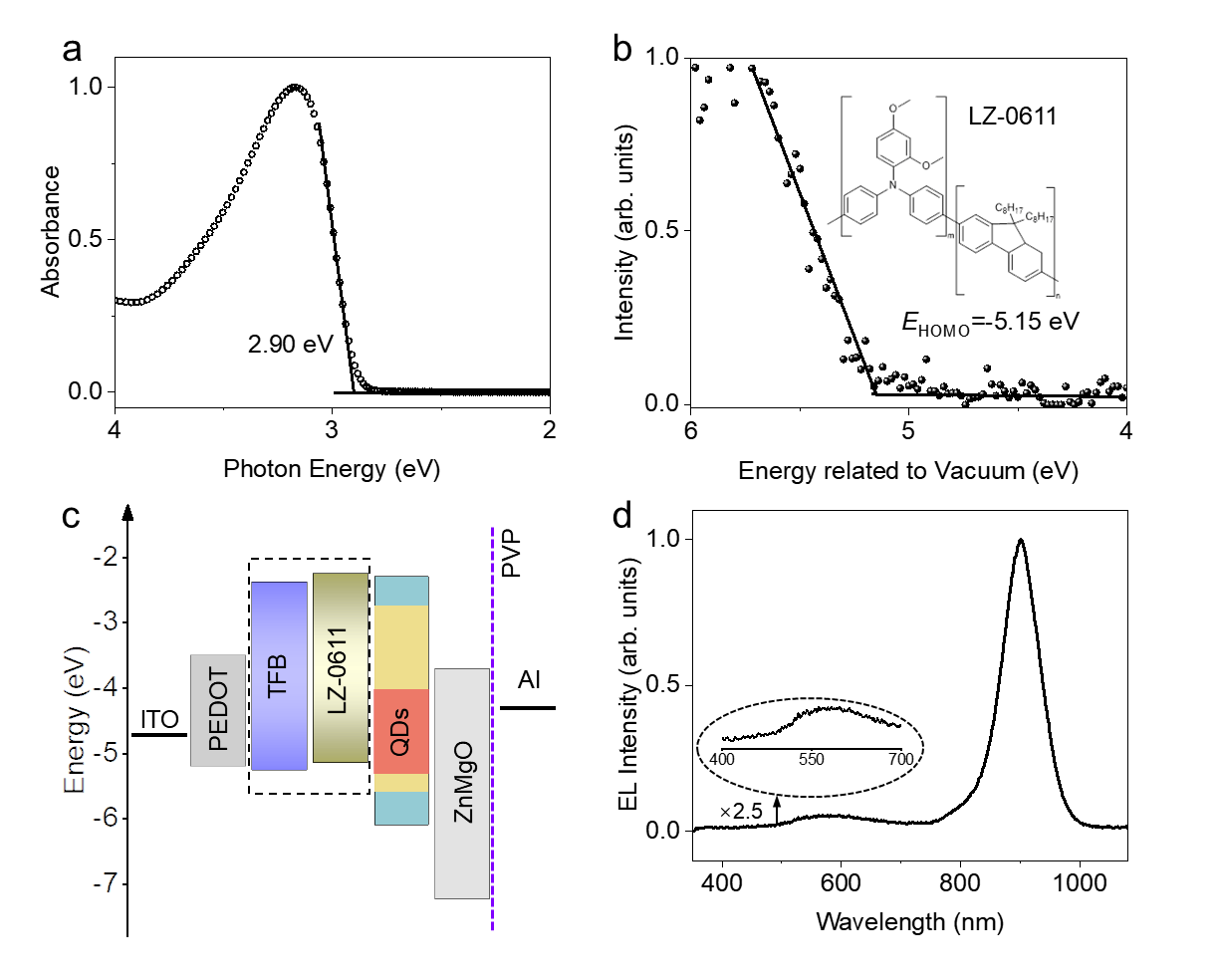
**

**Supplementary Fig. 24** **|** **Measurement of HOMO/LUMO energy levels of LZ-0611 and EL spectrum of QD-LEDs using LZ-0611 as the HTL. a** Absorption spectrum of a TFB-derived hole-transport polymer (LZ-0611, purchased from LinkZill company). **b** The HOMO energy of LZ-0611 measured by UPS. Inset: the molecular structure of LZ-0611. **c** Flat energy level diagram of QD-LEDs based on the HTLs of TFB or LZ-0611. **d** EL spectra of NIR QD-LEDs using LZ-0611 as the HTL. The applied voltage is 4.0 V. Evident parasitic emission from HTL is also observed in this device.


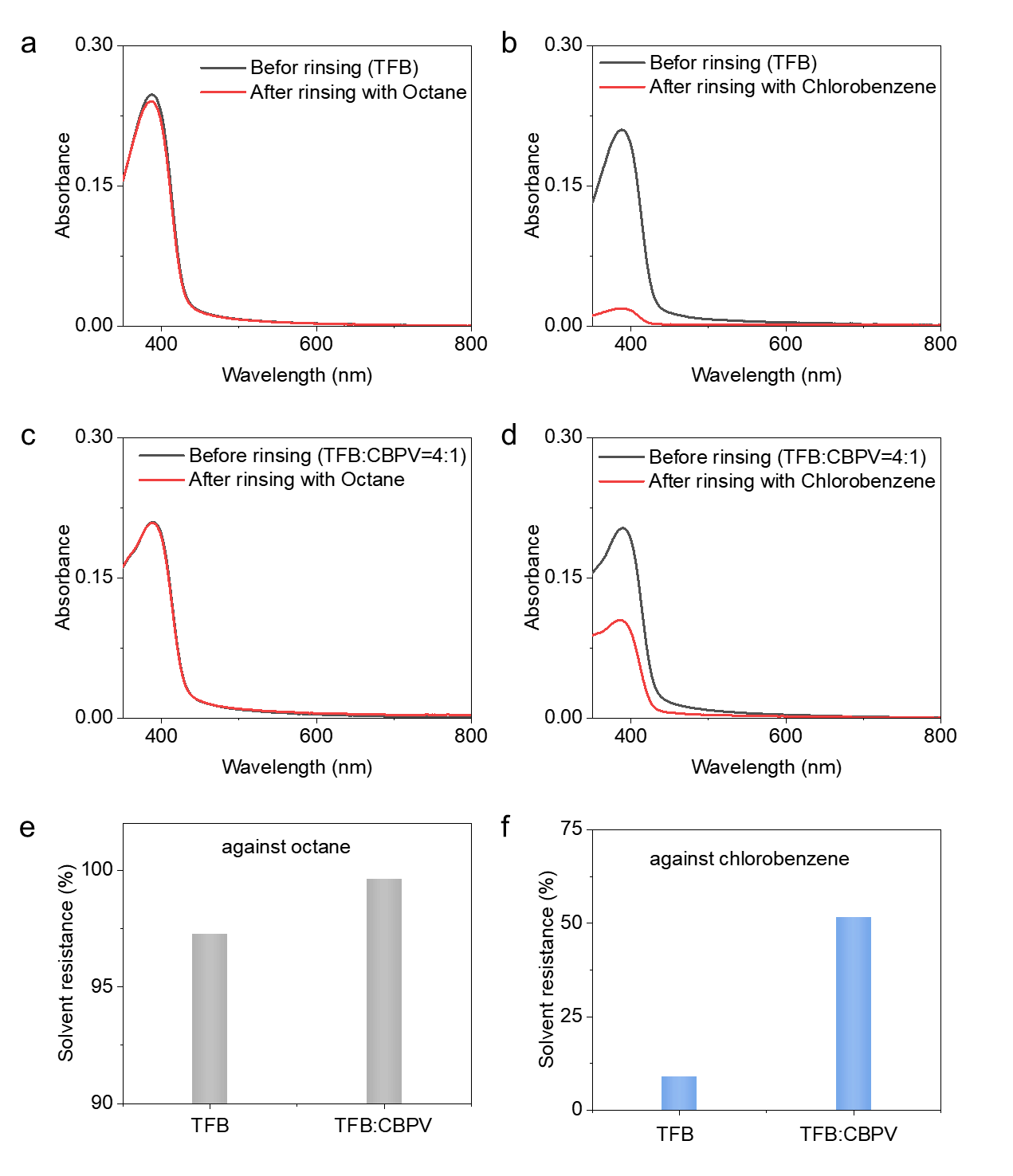


**Supplementary Fig. 25** **|** **Solvent resistance of TFB and crosslinked TFB:CBPV films.** **a,b** UV-vis absorption spectra of TFB film before (black) and after (red) rinsing with 40 μL of (**a**) octane or (**b**) chlorobenzene. **c,d** UV-vis absorption spectra of the crosslinked film (TFB:CBPV = 4:1) before (black) and after (red) rinsing with 40 μL of (**c**) octane (gray) and (**d**) chlorobenzene (blue). **e**,**f** Comparison of solvent resistance of TFB film and the crosslinked TFB:CBPV film against 40 μL of octane (**e**) and chlorobenzene (**f**).


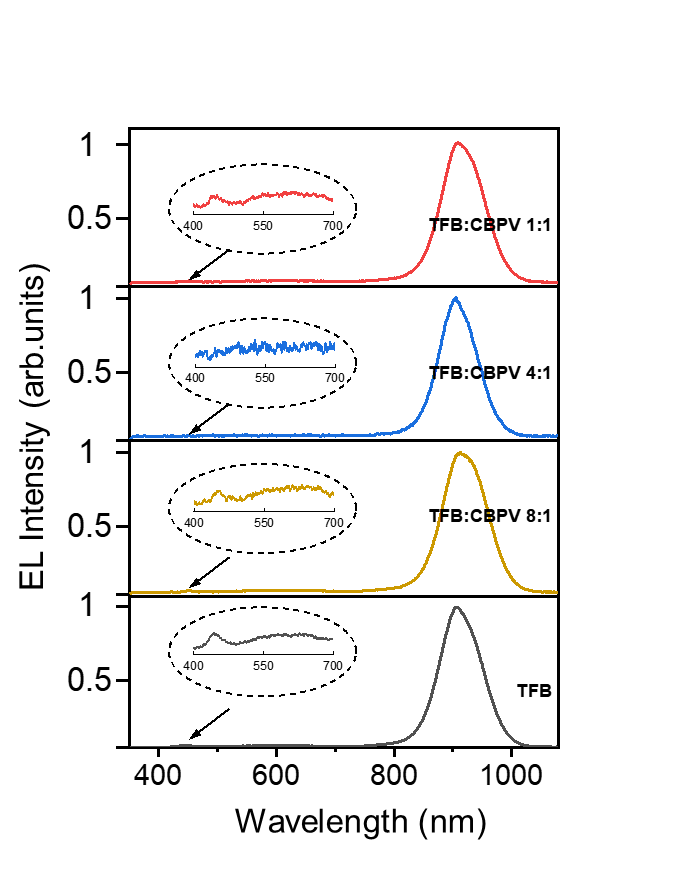


**Supplementary Fig. 26 |** **EL spectra of NIR QD-LEDs with varying CBPV amount in HTLs.** EL spectra of NIR QD-LEDs using TFB (black) and the crosslinked TFB:CBPV with different TFB and CBPV weight ratios (brown-yellow, 8:1; blue, 4:1; red, 1:1) as HTLs. The EL spectra between 400 and 700 nm are zoomed in to indicate the parasitic emissions from the HTLs.

**Supplementary Fig. 27** **|** **Voltage-dependent capacitance characteristics of QD-LEDs with varying CBPV amount in HTLs.** The voltage-dependent capacitance (CV) characteristics of QD-LEDs using TFB (black ) and the crosslinked TFB:CBPV with different TFB and CBPV ratios (red, 8:1; blue, 4:1; brown-yellow, 1:1) as HTLs. The modulating frequency is 1 kHz in the measurements. For TFB-based QD-LEDs, the capacitance increases rapidly at ~1.4 V and then decreases sharply when reaching the peak value of 2.6 nF at ~2.9 V, which signs the occurrence of effective hole transfer to QDs under high bias and following radiative recombination.


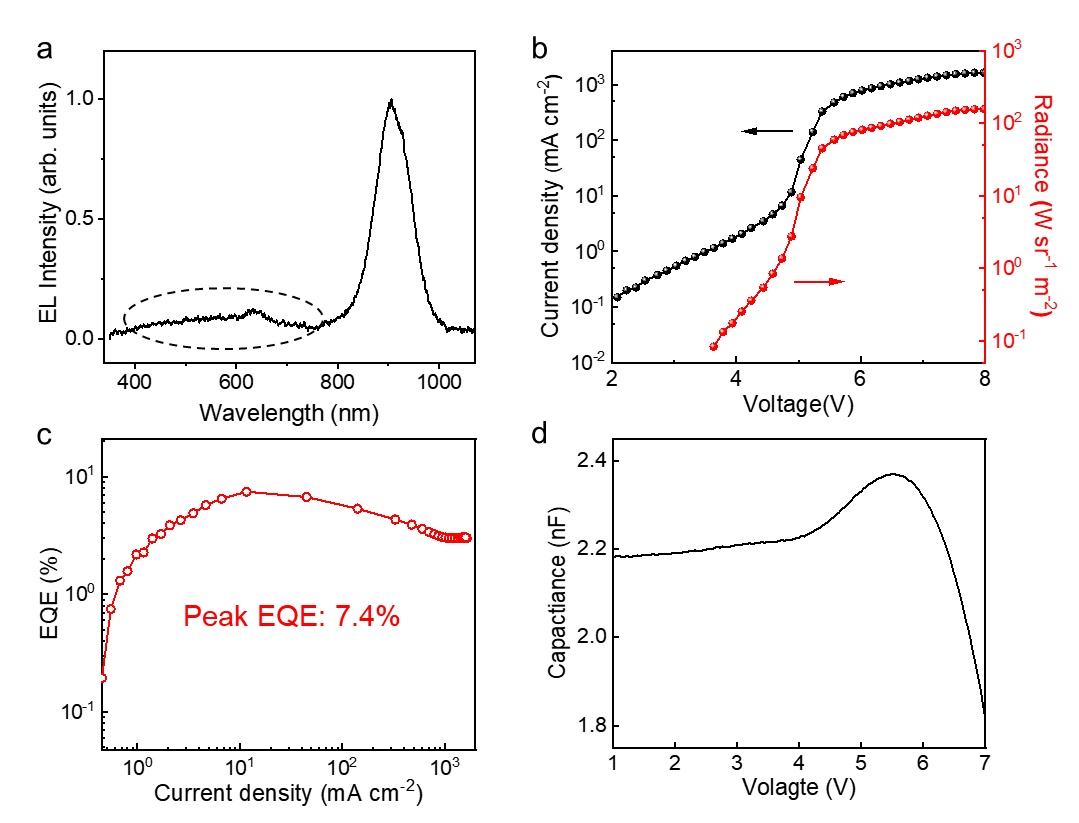


**Supplementary Fig. 28** **| Performance of NIR QD-LEDs using PVK as HTL. a** EL spectra of NIR QD-LEDs at an applied voltage of 8.0 V. The dashed ellipse marks the visible region parasitic emission from poly(9-vinylcarbazole) (PVK). **b** Current density (black) and radiance (red) of QD-LEDs as a function of voltage. **c** External quantum efficiency (EQE) of QD-LEDs as a function of voltage. The peak EQE is ~7.4 % at a driving voltage of 4.9 V (current density: 11.65 mA cm^-2^; radiance: 2.74 W sr^-1^ m^-2^). **d** Voltage-dependent capacitance (CV) characteristics of QD-LEDs.

**
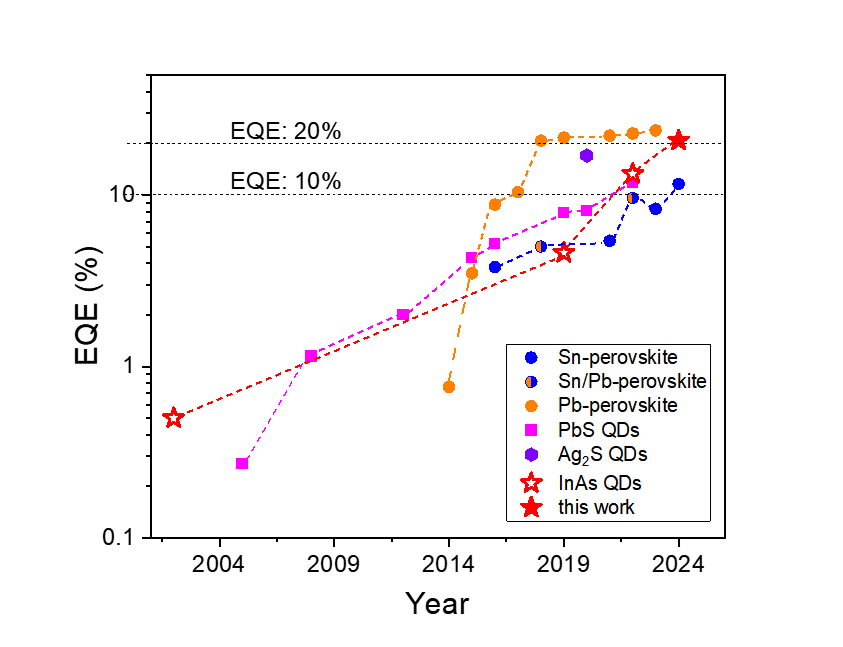
**

**Supplementary Fig. 29 |** **Evolution of EQE of a variety of solution-processed NIR LEDs along with time.** These NIR LEDs include Sn-based perovskite LEDs (blue solid circle), Sn/Pb-based perovskite LEDs (orange/blue solid circle), Pb-based perovskite LEDs (orange solid circle), PbS QDs-based LEDs (violet solid square), Ag_2_S QDs-based LEDs (violet solid hexagon), InAs QDs-based LEDs (red hollow pentagon). The external quantum efficiency (EQE) value of this work is marked as red solid pentagon. The two black dashed lines represent the 10% and 20% EQE values, respectively.


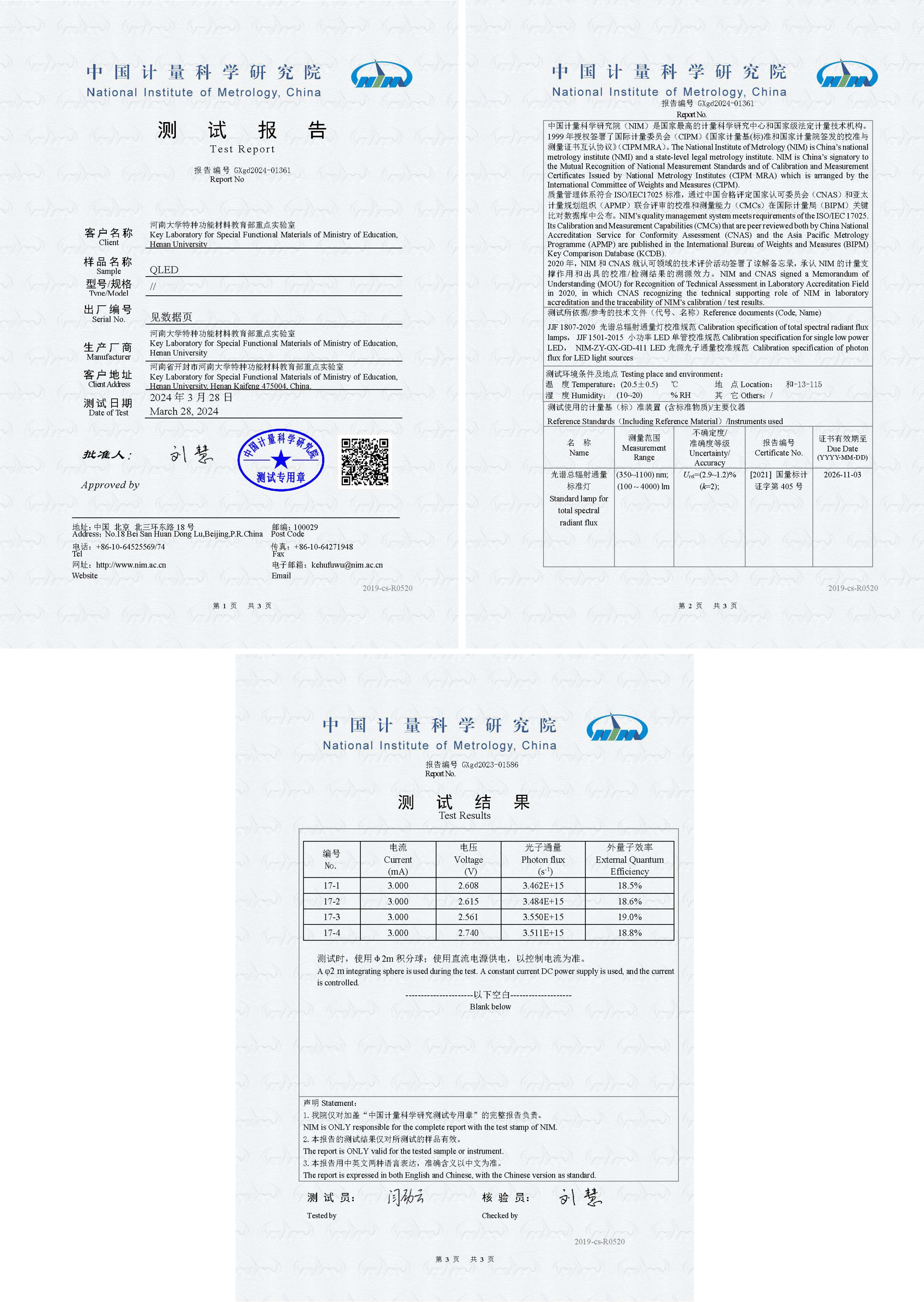


**Supplementary Fig. 30** **|** **Certified EQE of our NIR QD-LEDs from National Institute of Metrology of China.** The EQE were measured using a φ2 m integrating sphere. The peak EQE of the certified NIR QD-LEDs device is 19.0%. Photons trapped within the glass substrate are not gathered.


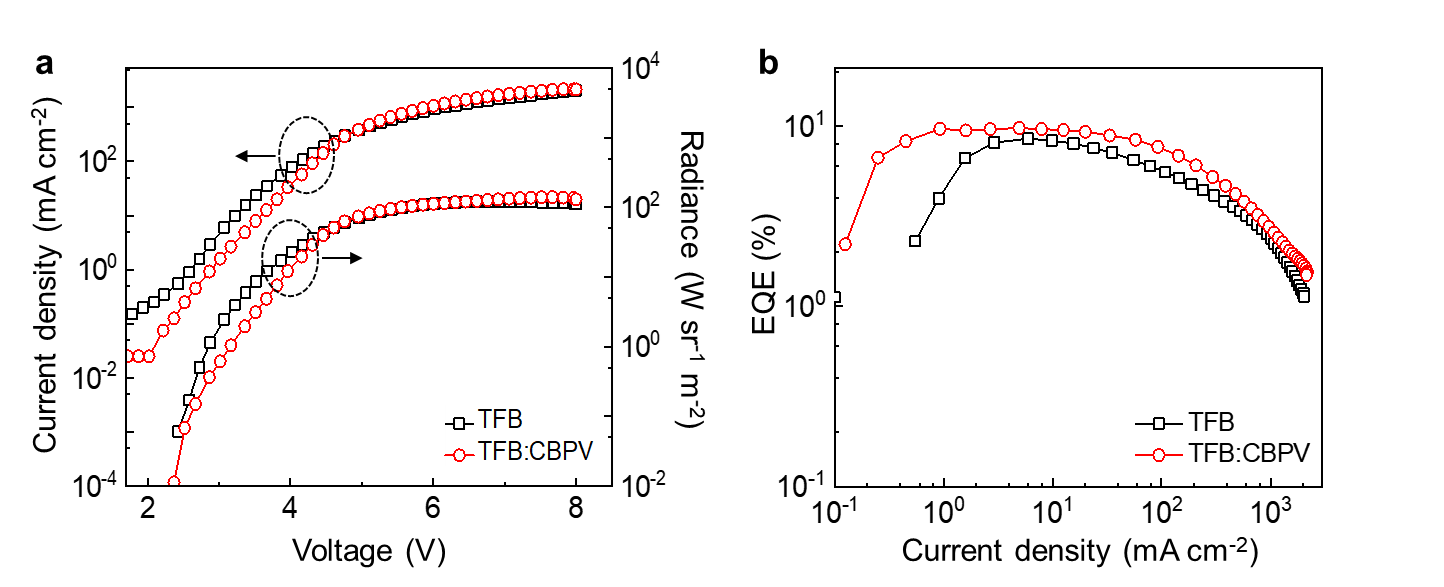


**Supplementary Fig. 31** **|** **Comparative performance of NIR QD-LEDs based on TFB and crosslinked TFB:CBPV HTLs when using untreated QDs as emitters.** **a** Current density (left) and radiance (right) as a function of voltage for NIR QD-LEDs based on TFB HTLs (black) and TFB:CBPV HTLs (red). **b** EQE as a function of voltage for NIR QD-LEDs based on TFB HTL (black) and TFB:CBPV HTL (red). The peak EQE values of QD-LEDs based on TFB and TFB:CBPV as HTLs are 8.5% and 9.8%, respectively.


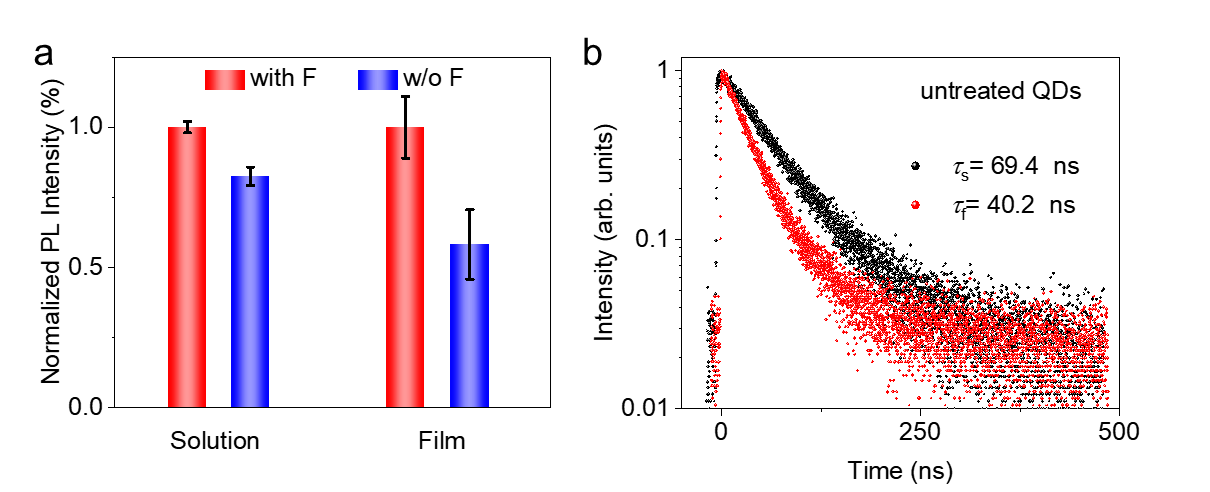


**Supplementary Fig. 32 | Comparison of optical properties of QDs synthesized with and without ZnF_2_. a** Comparison of normalized photoluminescence (PL) intensity of ZnF_2_-treated QDs (with F, red) and untreated QDs (w/o F, blue). Left: normalized PL intensity of QDs in solution; Right: normalized PL intensity of QDs films. The PL intensity of ZnF_2_-treated QDs is normalized to 1. The error bars of PL intensity in (**a**) represent the standard deviation of multiple syntheses results with respect to the mean values. The relative PL intensity of untreated QDs is normalized based on the absorption intensity at the excitation wavelength. **b** Time-resolved PL decay curves of untreated QDs dispersed in toluene (black) and deposited as films on glass (red), showing a solution lifetime (*τ*_s_ ​) of 69.4 ns and a film lifetime (*τ*_f_) of 40.2 ns. The Förster resonance energy transfer (FRET) efficiency of the untreated QDs film is calculated to be 42.1%. When QDs are deposited as dense arrays, some poor-emitting QDs act as channels for non-radiative recombination, where FRET can occur efficiently. The density of non-radiative centers in untreated QDs films is expected to be larger than in films from ZnF_2_-treated QDs with near-unity PLQY, causing more severe FRET-induced PL quenching in the former.

**Supplementary Table 1. Summary of solution-processed NIR LEDs with high performance in terms of EQE, radiance and lifetime** The lifetime *T*_50_ at 50 W sr^-1^ m^-2^ was calculated by using the relation *L*_0_^n^T = Constant, where n is acceleration factor. If not reported in the literatures, the acceleration factor (n) will be adopted to be 2 or 1.5 for the tested initial radiance larger or smaller than 50 W sr^−1^ m^−2^, respectively, so that the calculated *T*_50_ at 50 W sr^-1^ m^-2^ is generally overestimated.

|  | **Structure** | **Peak Radiance**  **W sr^-1^ m^-2^** | **Peak EQE**  **(%)** | **EL**  **(nm)** | **Half-lifetime *T*_50_ (h)** | | | | **Supplementary Reference** |
| --- | --- | --- | --- | --- | --- | --- | --- | --- | --- |
|  |  |  |  |  | ***T*_50_ (h) @**  **_W sr^-1^ m^-2^** | **n** | ***T*_50_ (h) @**  **50 W sr^-1^ m^-2^** | **Current Density (mA m^-2^)** |  |
| Sn-  perovskite | ITO/PVK/PEAI-CsSnI_3_/TMPyPb/LiF/Al | 40 | 3.0 | 920 | 2 h@0.6 | 1.5 | 0.03 | 10 | 2019 J. Phys. Chem. Lett.^2^ |
|  | ITO/PEDOT/CsSnI_3_/PBD/LiF/Al | 40 | 3.8 | 950 | NA | NA | NA | NA | 2016 Adv. Mater.^3^ |
|  | ITO/PEDOT/VASC+PH-FASnI_3_/ TMPyPb/LiF/Al | 10.8^†^ | 5.3 | 866 | 0.25 h@1^†^ | 1.5 | ＜0.01 | 10 | 2022 Adv. Mater.^4^ |
|  | ITO/PEDOT:PSS/CsSnI_3_/  B3PYMPM/LiF/Al | 162 | 5.4 | 932 | 23.6 h@162 | 2 | 247.7 | 100 | 2021 Adv. Mater.^5^ |
|  | ITO/PEDOT:PSS/CsSnI_3_-FASCN/B3PYMPM/LiF/Al | 20^†^ | 5.3 | 956 | NA | NA | NA | NA | 2023 ACS Energy Lett.^6^ |
|  | ITO/PEDOT:PSS/FA_0.9_Cs_0.1_SnI_3_/  TBPi/LiF/Al | 12 | 8.3 | 894 | 3 h@12^❊^ | 1.5 | 0.35 | 1 | 2023 Nat. Photonics^7^ |
|  | ITO/PEDOT:PSS/ CsSnI_3_/  TBPi/LiF/Al | 226 | 3.2 | 948 | 39.5@18^†^ | 1.5 | 8.5 | 100 | 2024 Nat. Photonics^8^ |
|  | ITO/PEDOT:PSS/  2D-3D Sn perovskite/TBPi/LiF/Al | 89 | 11.6 | 898 | 0.38@66.7^†^ | 2 | 0.68 | 100 | 2024 Nat. Nanotechnol.^9^ |
| Sn/Pb-  perovskite | ITO/PolyTPD/FPMAI-MAPb_0.6_Sn_0.4_I_3_/TPBi/LiF/Al | 2.7 | 5.0 | 917 | NA | NA | NA | NA | 2019 Adv. Mater.^10^ |
|  | ITO/Poly-TPD/KI-MAPb_0.8_Sn_0.2_I_3_/TPBi/LiF/Al | 2.5^†^ | 9.6 | 868 | NA | NA | NA | NA | 2022 Sci. Bull.^11^ |
| Pb-  perovskite | ITO/TiO_2_/Al_2_O_3_(1nm)/  MAPbI_3–x_Cl_x_/F8/MoO_3_/Ag | 13.2 | 0.76 | 754 | NA | NA | NA | NA | 2014 Nat. Nanotechnol.^12^ |
|  | ITO/TiO_2_/PEA_2_(MA)_4_Pb_4_I_13_ /F8/MoO_3_/Au | 80 | 8.8 | 750 | NA | NA | NA | NA | 2016 Nat. Nanotechnol.^13^ |
|  | ITO/ZnO/PEIE/  MAPbI_3-x_Cl_x_/TFB/MoO_x_/Au | 28 | 3.5 | 768 | NA | NA | NA | NA | 2015 Adv. Mater.^14^ |
|  | ITO/ZnO/2D-3D PVSK/  Poly-TPD/MoO_3_/Ag | 191.5 | 7.7 | 812 | 202.7@10 | 1.5 | 18.1 | 100^†^ | 2020 Adv. Mater.^15^ |
|  | ITO/PolyTPD/MAPbI_3_/  TPBi/LiF/Al | NA | 10.4 | 748 | NA | NA | NA | NA | 2017 Nat. Photonics^16^ |
|  | ITO/ZnO/PEIE/FAPbI_3_/  TFB/MoO_x_/Au | 241 | 14.2 | 804 | 23.7 h@ 67 | 2 | 42.6 | 100 | 2019 Nat. Commun.^17^ |
|  | ITO/ZnO/PEIE/PPAI-FA_0.83_Cs_0.17_PbI_3_/TFB/Au | 1282.8 | 17.5 | 789 | 130 h @70^†^ | 2 | 254.8 | 100 | 2021 Nat. Commun.^18^ |
|  | ITO/ZnO/PEIE/FAPbI_3_-5AVA/Poly-TPD /MoO_x_/Au | 390 | 20.7 | 803 | 20 h @100^†^ | 2 | 80.0 | 100 | 2018 Nature^19^ |
|  | ITO/ZnO/PEIE/FAPbI_3_/FTB  /MoO_3_/Au | 308 | 21.6 | 800 | 0.3 h@ 100^†^ | 2 | 1.2 | 200 | 2019 Nat. Photonics^20^ |
|  | ITO/ZnO/PEIE/FAPbI_3_-AEAA/TFB/MoO_x_/Au | 250^†^ | 22.2 | 800 | 18.6 h@ 100^†^ | 2 | 74.4 | 100 | 2021 Nat. Commun.^21^ |
|  | ITO/ZnO/PEIE/FAPbI_3_-SFB10/TFB/MoO_x_/Au | 278.9 | 22.8 | 800 | 14.1^††^@118.8 | 2 | 79.6 | 200 | 2022 Nat. Photonics^22^ |
|  | ITO/ZnO/PEIE/FAPbI_3_-MSPE/Poly-TPD/MoO_x_/Au | 497 | 23.8 | 800 | 32 h@107 | 2 | 146.5 | 100 | 2023 Nature^23^ |
|  | ITO/ZnO-PEIE/FAPbI_3_ /TFB/MoO_x_/Au | 390 | 32.0 | 805 | 17h@140.9^†^ | 2 | 135.0 | 100 | 2024 Nature^24^ |
| PbS  QDs | ITO/MEH-PPPV PbS/Al:Li/Ag | NA | ＜0.01 | 1350 | NA | NA | NA | NA | 2003 Appl. Phys. Lett.^25^ |
|  | ITO/PPV/MEH-PPV/PbS/Mg/Ag | NA | 0.5 | 1160 | NA | NA | NA | NA | 2005 Adv. Funct. Mater.^26^ |
|  | ITO/Pentacene/nc-PbS/BCP/Al | NA | 1.15 | 1200 | NA | NA | NA | NA | 2008 Appl. Phys. Lett.^27^ |
|  | ITO/PEDOT/PbS/ZnO/Al | 6.4 | 2.0 | 1232 | NA | NA | NA | NA | 2012 Nat. Nanotechnol.^28^ |
|  | ITO/ZnO/PbS-CdS QDs/CBP/MoO_3_/Au | 0.75^†^ | 4.3 | 1242 | NA | NA | NA | NA | 2015 Adv. Mater.^29^ |
|  | ITO/TiO_2_/PbS-PVK Matrix/F8/MoO_3_/Ag | 0.92 | 5.2 | 1391 | NA | NA | NA | NA | 2016 Nat. Photonics^30^ |
|  | ITO/ZnO/ZnO-PbS(donor)-PbS(emitter)/PbS(Blocking)/Au | 9 | 7.9 | 1400 | 48 h@9 | 1.5 | 3.6 | 1.75 | 2019 Nat. Nanotechnol.^31^ |
|  | ITO/ZnO/PbS(Matrix)/  PbS(Emitter)/ PbS(Blocking)/Au | 35 | 8.1 | 1400 | 532 h@7 | 1.5 | 27.9 | 10^†^ | 2020 Adv. Funct. Mater.^32^ |
|  | ITO/PEDOT:PSS/QDLP  /TPBi/LiF/Al | 7.4 | 8.1 | 980 | 1.2^†^@0.01^†^ | 1.5 | ＜0.01 | 10 | 2020 Nat. Photonics^33^ |
|  | ITO/ZnO/PbS(Active layer)/PbS  (Blocking layer)/Au | 10 | 11.8 | 1550 | NA | NA | NA | NA | 2022 Adv. Sci.^34^ |
| Ag_2_S  QDs | ITO/TiO_2_/Ag_2_S@SiO_2_ in Perovskite/Porphyrin/MoO_3_/Al | 83.9 | 16.98 | 1397 | 480 h@83(*T*_85_) | 2 | 1322.7 (*T*_85_) | 10^†^ | 2020 Nat. Photonics^35^ |
| Pt (II)  Complex | ITO/MoO_3_/NPB/Pt-27-d/CN-T2T/CN-T2T:Liq/Al | 60^†^ | 5.17 | 930 | 460 h^†^@4^†^ | 1.5 | 10.4 | NA | 2022 Nat. Photonics^36^ |
| InAs  QDs | MEH-PPV/InAs-ZnSe | NA | 0.5 | 1270 | NA | NA | NA | NA | 2002 Science^37^ |
|  | ITO/ZnO/PEIE/QDs/  Poly-TPD/MoO_3_/Al | 8.2 | 4.6 | 857 | 20 h @8.2^❊^(*T*_85_) | 1.5 | 1.3 (*T*_85_) | 2^†^ | 2019 Adv. Funct. Mater.^38^ |
|  | ITO/PEDOT:PSS/Poly-TPD/  InAs/ZnSe QDs/TPBi/LiF/Al | 0.15 | 5.5 | 947 | 32 h @0.15 | 1.5 | ＜0.01 | 0.1 | 2022 ACS Energy Lett.^39^ |
|  | ITO/ZnO/ Poly(9-vinylcarbazole) /PEIE/CQDs/Poly-TPD/ MoO_3_/Al | 2.2 | 13.3 | 1033 | 20 h @2.2^❊^(*T*_85_) | 1.5 | 0.2 (*T*_85_) | 0.05^†^ | 2022 Adv. Mater.^40^ |
|  | ITO/ZnO/PMMA/InAs/ZnSe QDs/ Poly-TPD/MoO_x_/Al | 12 | 13.3 | 900 | 0.8@0.1 | 1.5 | ＜0.01 | ＜0.5 | 2024 Adv. Sci.^41^ |
|  | ITO/PEDOT:PSS/TFB:CBPV/QDs/ZnMgO/Al | 581.4 | 20.4 | 905 | 550 h@50 | / | 550 | 36.3 | This work |

^†^Estimated data from the figure in the references.

^††^Average lifetime in the references.

^❊^Assuming the peak radiance as initial radiance value.

**Supplementary References**

1. Reiss, P., Protière, M., Li, L. Core/shell semiconductor nanocrystals. *Small* **5**, 154-168 (2009).

2. Wang, Y. *et al.* Tin-based multiple quantum well perovskites for light-emitting diodes with improved stability. *J. Phys. Chem. Lett.* **10**, 453-459 (2019).

3. Hong, W.-L. *et al.* Efficient low-temperature solution-processed lead-free perovskite infrared light-emitting diodes. *Adv. Mater.* **28**, 8029-8036 (2016).

4. Zhang, F. *et al.* Vapor-assisted in situ recrystallization for efficient tin-based perovskite light-emitting diodes. *Adv. Mater.* **34**, 2203180 (2022).

5. Lu, J. *et al.* Dendritic CsSnI_3_ for efficient and flexible near-infrared perovskite light-emitting diodes. *Adv. Mater.* **33**, 2104414 (2021).

6. Guan, X. *et al.* Suppressing disproportionation decomposition in Sn-based perovskite light-emitting diodes. *ACS Energy Lett.* **8**, 1597-1605 (2023).

7. Min, H. *et al.* Additive treatment yields high-performance lead-free perovskite light-emitting diodes. *Nat. Photonics* **17**, 755-760 (2023).

8. Yuan, F., *et al.* Bright and stable near-infrared lead-free perovskite light-emitting diodes. *Nat. Photonics* **18**, 170–176 (2024).

9. Min, H. *et al.* Spin coating epitaxial heterodimensional tin perovskites for light-emitting diodes. *Nat. Nanotechnol.* (2024).

10. Qiu, W. *et al.* Mixed lead–tin halide perovskites for efficient and wavelength-tunable near-infrared light-emitting diodes. *Adv. Mater.* **31**, 1806105 (2019).

11. Yu, H. *et al.* Alkalis-doping of mixed tin-lead perovskites for efficient near-infrared light-emitting diodes. *Sci. Bull.* **67**, 54-60 (2022).

12. Tan, Z.-K. *et al.* Bright light-emitting diodes based on organometal halide perovskite. *Nat. Nanotechnol.* **9**, 687-692 (2014).

13. Yuan, M. *et al.* Perovskite energy funnels for efficient light-emitting diodes. *Nat. Nanotechnol.* **11**, 872-877 (2016).

14. Wang, J. *et al.* Interfacial control toward efficient and low-voltage perovskite light-emitting diodes. *Adv. Mater.* **27**, 2311-2316 (2015).

15. Han, T.-H. *et al.* Surface-2d/bulk-3d heterophased perovskite nanograins for long-term-stable light-emitting diodes. *Adv. Mater.* **32**, 1905674 (2020).

16. Xiao, Z. *et al.* Efficient perovskite light-emitting diodes featuring nanometre-sized crystallites. *Nat. Photonics* **11**, 108-115 (2017).

17. Miao, Y. *et al.* Stable and bright formamidinium-based perovskite light-emitting diodes with high energy conversion efficiency. *Nat. Commun.* **10**, 3624 (2019).

18. Guo, Y. *et al.* Phenylalkylammonium passivation enables perovskite light emitting diodes with record high-radiance operational lifetime: the chain length matters. *Nat. Commun.* **12**, 644 (2021).

19. Cao, Y. *et al.* Perovskite light-emitting diodes based on spontaneously formed submicrometre-scale structures. *Nature* **562**, 249-253 (2018).

20. Xu, W. *et al.* Rational molecular passivation for high-performance perovskite light-emitting diodes. *Nat. Photonics* **13**, 418-424 (2019).

21. Zhu, L. *et al.* Unveiling the additive-assisted oriented growth of perovskite crystallite for high performance light-emitting diodes. *Nat. Commun.* **12**, 5081 (2021).

22. Guo, B. *et al.* Ultrastable near-infrared perovskite light-emitting diodes. *Nat. Photonics* **16**, 637-643 (2022).

23. Sun, Y. *et al.* Bright and stable perovskite light-emitting diodes in the near-infrared range. *Nature* **615**, 830-835 (2023).

24. Li, M. *et al.* Acceleration of radiative recombination for efficient perovskite LEDs. *Nature.* **630**, 631-635 (2024).

25. Bakueva, L. *et al.* Size-tunable infrared (1000–1600 nm) electroluminescence from PbS quantum-dot nanocrystals in a semiconducting polymer. *Appl. Phys. Lett.* **82**, 2895-2897 (2003).

26. Konstantatos, G., Huang, C., Levina, L., Lu, Z., Sargent, E. H. Efficient infrared electroluminescent devices using solution-processed colloidal quantum dots. *Adv. Funct. Mater.* **15**, 1865-1869 (2005).

27. Bourdakos, K. N., Dissanayake, D. M. N. M., Lutz, T., Silva, S. R. P., Curry, R. J. Highly efficient near-infrared hybrid organic-inorganic nanocrystal electroluminescence device. *Appl. Phys. Lett.* **92**, 153311 (2008).

28. Sun, L. *et al.* Bright infrared quantum-dot light-emitting diodes through inter-dot spacing control. *Nat. Nanotechnol.* **7**, 369-373 (2012).

29. Supran, G. J. *et al.* High-performance shortwave-infrared light-emitting devices using core–shell (PbS–CdS) colloidal quantum dots. *Adv. Mater.* **27**, 1437-1442 (2015).

30. Gong, X. *et al.* Highly efficient quantum dot near-infrared light-emitting diodes. *Nat. Photonics* **10**, 253-257 (2016).

31. Pradhan, S. *et al.* High-efficiency colloidal quantum dot infrared light-emitting diodes via engineering at the supra-nanocrystalline level. *Nat. Nanotechnol.* **14**, 72-79 (2019).

32. Pradhan, S., Dalmases, M., Baspinar, A.-B., Konstantatos, G. Highly efficient, bright, and stable colloidal quantum dot short-wave infrared light-emitting diodes. *Adv. Funct. Mater.* **30**, 2004445 (2020).

33. Gao, L. *et al.* Efficient near-infrared light-emitting diodes based on quantum dots in layered perovskite. *Nat. Photonics* **14**, 227-233 (2020).

34. Pradhan, S., Dalmases, M., Taghipour, N., Kundu, B., Konstantatos, G. Colloidal quantum dot light emitting diodes at telecom wavelength with 18% quantum efficiency and over 1 MHz bandwidth. *Adv. Sci.* **9**, 2200637 (2022).

35. Vasilopoulou, M. *et al.* Efficient colloidal quantum dot light-emitting diodes operating in the second near-infrared biological window. *Nat. Photonics* **14**, 50-56 (2020).

36. Tessler, N., Medvedev, V., Kazes, M., Kan, S., Banin, U. Efficient near-infrared polymer nanocrystal light-emitting diodes. *Science* **295**, 1506-1508 (2002).

37. Wang, SF. *et al.* Polyatomic molecules with emission quantum yields >20% enable efficient organic light-emitting diodes in the NIR(II) window. *Nat. Photonics* 16, 843-850 (2022).

38. Wijaya, H. *et al.* Efficient near-infrared light-emitting diodes based on In(Zn)As–In(Zn)P–GaP–ZnS quantum dots. *Adv. Funct. Mater.* **30**, 1906483 (2020).

39. De Franco, M. *et al.* Near-infrared light-emitting diodes based on RoHS-compliant InAs/ZnSe colloidal quantum dots. *ACS Energy Lett.* **7**, 3788-3790 (2022).

40. Zhao, X., Lim, L. J., Ang, S. S., Tan, Z.-K. Efficient short-wave infrared light-emitting diodes based on heavy-metal-free quantum dots. *Adv. Mater.* **34**, 2206409 (2022).

41. Roshan, H. *et al.* Near infrared light-emitting diodes based on colloidal InAs/ZnSe Core/thick-shell quantum dots. *Adv. Sci.* 22400734 (2024).
