# Supplementary material for: Efficient and stable near-infrared InAs quantum dot light-emitting diodes
Source: Nat Commun. 2025 Mar 12;16:2450. doi: 10.1038/s41467-025-57746-1 (PMC11897344; doi:10.1038/s41467-025-57746-1)
Supplement: Supplementary file 2 — Transparent Peer Review file [file 41467_2025_57746_MOESM2_ESM.pdf]

nature portfolio

Peer Review File

Efficient and stable near-infrared InAs quantum dot light-emitting diodes

Corresponding Author: Professor Botao Ji

This manuscript has been previously reviewed at another journal. This document only contains information relating to versions considered at Nature Communications.

This file contains all reviewer reports in order by version, followed by all author rebuttals in order by version.

Version 0:

Reviewer comments:

Reviewer #1

(Remarks to the Author)
The revisions are satisfactory. I recommend the acceptance of this manuscript for publication at Nature Comm.

Reviewer #2

(Remarks to the Author)
The authors have addressed all my questions carefully, and I look forward to the publication of the ms.

Open Access This Peer Review File is licensed under a Creative Commons Attribution 4.0 International License, which permits use, sharing, adaptation, distribution and reproduction in any medium or format, as long as you give appropriate credit to the original author(s) and the source, provide a link to the Creative Commons license, and indicate if changes were made.

In cases where reviewers are anonymous, credit should be given to 'Anonymous Referee' and the source.
The images or other third party material in this Peer Review File are included in the article's Creative Commons license, unless indicated otherwise in a credit line to the material. If material is not included in the article's Creative Commons license and your intended use is not permitted by statutory regulation or exceeds the permitted use, you will need to obtain permission directly from the copyright holder.
To view a copy of this license, visit https://creativecommons.org/licenses/by/4.0/
